# Molecular mechanism of CCDC106 regulating the p53-Mdm2/MdmX signaling axis

**DOI:** 10.1038/s41598-023-47808-z

**Published:** 2023-12-11

**Authors:** Ting Zhou, Zhiqiang Ke, Qianqian Ma, Jiani Xiang, Meng Gao, Yongqi Huang, Xiyao Cheng, Zhengding Su

**Affiliations:** 1 School of Light Industry and Food Engineering, Guangxi University, No. 100, Daxuedong Road, Xixiangtang District, Nanning, 530004 Guangxi China; 2 Protein Engineering and Biopharmaceutical Sciences Group, Hubei University of Technology, Wuhan, 430068 China; 3 Hubei Key Laboratory of Diabetes and Angiopathy, Xianning Medical College, Hubei University of Science and Technology, Xianning, 437100 Hubei China

**Keywords:** Biochemistry, Cancer, Cell biology, Drug discovery, Molecular biology, Structural biology

## Abstract

The tumor suppressor p53 (p53) is regulated by murine double minute 2 (Mdm2) and its homologous MdmX in maintaining the basal level of p53. Overexpressed Mdm2/MdmX inhibits cellular p53 activity, which is highly relevant to cancer occurrence. Coiled-coil domain-containing protein 106 (CCDC106) has been identified as a p53-interacting partner. However, the molecular mechanism of the p53/Mdm2/MdmX/CCDC106 interactions is still elusive. Here, we show that CCDC106 functions as a signaling regulator of the p53-Mdm2/MdmX axis. We identified that CCDC106 directly interacts with the p53 transactivation domain by competing with Mdm2 and MdmX. CCDC106 overexpression downregulates the cellular level of p53 and Mdm2/MdmX, and decreased p53 reversibly downregulates the cellular level of CCDC106. Our work provides a molecular mechanism by which CCDC106 regulates the cellular levels of p53 and Mdm2/MdmX.

## Introduction

Endogenous CCDC106 and p53 can be colocalized in nuclei and interact with each other in vivo, promoting the degradation of the p53 protein and inhibiting its transactivation activity^1^, suggesting that CCDC106 is a negative regulator of p53 to promote cell proliferation in cancers. In ovarian cancers, the overexpression of CCDC106 promotes cell proliferation and invasion by suppressing p21 transcription through a p53-independent pathway, while CCDC106 knockdown inhibits the expression of proliferation, invasion and EMT signaling markers in mutant p53 cells but not in wild-type p53 cells^2^. During cancer progression, the phosphorylation of CCDC106 by the protein kinase CK2 is essential for p53 degradation, and a CK2 inhibitor can block the translocation of CCDC106 into the nuclei of mutant p53 cells^3^. In non-small cell lung cancer (NSCLC), the overexpression of CCDC106 significantly correlates with advanced TNM stage^4^. In two typical NSCLC cell lines in which H1299 cells overexpress CCDC106 but A549 cells express less CCDC106, the expression of CCDC106 upregulates the expression of Cyclin A2 and Cyclin B1, promoting cell proliferation via an Akt-dependent signaling pathway^4^.

Dysfunction of p53 is a key cause of cancer development^5^, while CCDC106 can reduce p53 stability^3^, suggesting that CCDC106 may be a novel target for cancer treatment. On the other hand, the phosphorylation of Mdm2 allows its entry into the nucleus, where it targets p53 for degradation^6,7^. Thus, it is likely that the interaction of CCDC106 with the p53-Mdm2/MdmX signaling pathway plays an important role in cancer cell survival and drug resistance. However, it is elusive how CCDC106 signaling regulates the p53 signaling pathway. Here, we identified that CCDC106 competes with Mdm2 and MdmX and directly interacts with the transactivation domain of p53. Such interaction downregulates p53 and Mdm2, promoting cell proliferation. Thus, our work reveals a structural mechanism by which CCDC106 interacts with p53.

## Results

### Characterization of the CCDC106 protein structure bioinformatically

**Figure 1:**
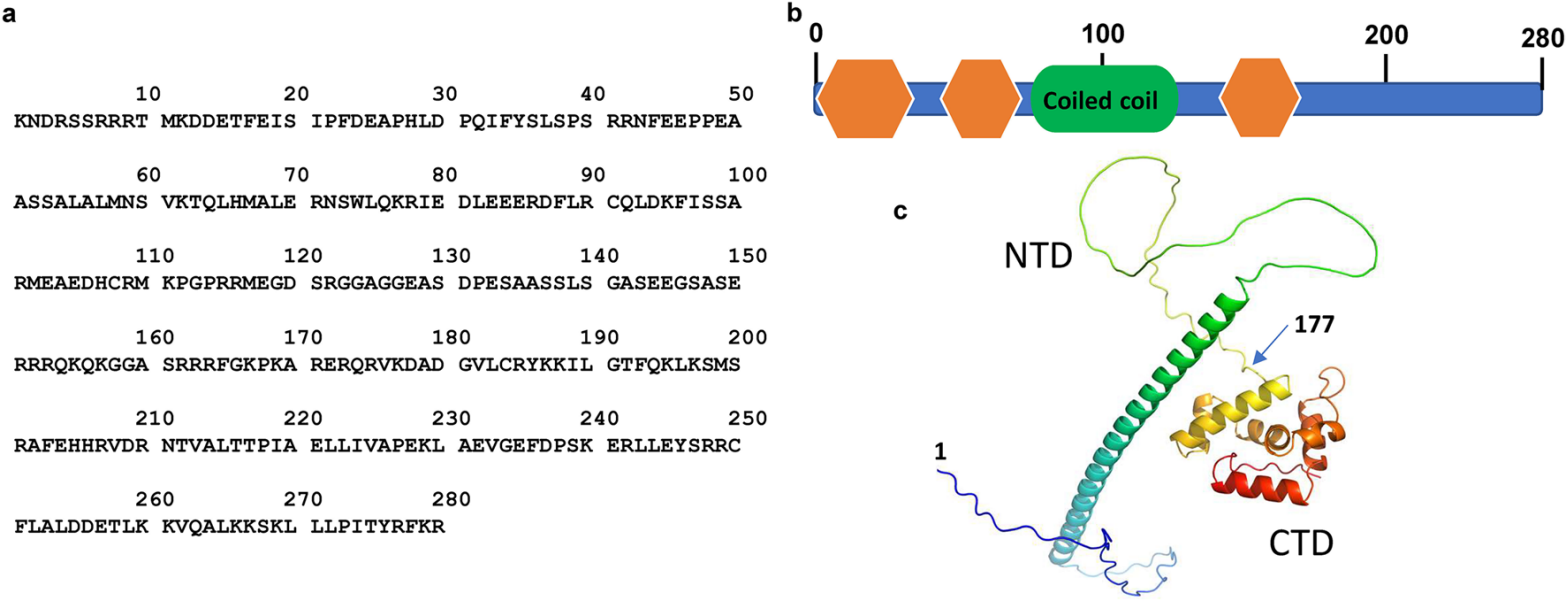
Prediction of the structural domains of the CCDC106 protein. (**a**) Amine acid sequence of human CCDC106. (**b**) Potential domains in CCDC106 predicted by the SMART program. Putative domains are described in the *Supplementary Information*. (**c**) The putative three-dimensional structure of CCDC106 was predicted with the alphaFold2 program. Source data for (**c**) are provided as a Source Data file.

CCDC106 is a single polypeptide with 280 amino acid residues (Fig. 1a). We predicted its putative functional domains using the Simple Modular Architecture Research Tool (SMART)^8^. As shown in Fig. 1b, it contains a coiled coil domain predicted with confidence (Table S1). However, it is also likely that CCDC106 may have the potential to constitute many other functional domains (Table S2), although their e-values are over threshed. As predicted with the alphaFold2 program, the CCDC106 structure contains a long α-helix flanked by two coils at the two ends of the α-helix in the N-terminal region of the CCDC106 protein, while its C-terminal region forms a compact helix-rich structure. Therefore, we arbitrarily defined these two regions as the N-terminal domain (NTD) and the C-terminal domain (CTD) of CCDC106, respectively (Fig. 1c).

To understand how CCDC106 interacts with the p53 protein, we recombinantly expressed full-length CCDC106 with FLAG and GFP tags in mammalian cells, while its NTD and CTD proteins were expressed in GST fusion form using *E. coli* cells (Supplementary Fig. S1). These two GST fusion proteins were used as baits to pull down p53 to investigate the interaction between CCDC106 and p53.

### The expression of cellular CCDC106 is correlated with the cellular p53 level

**Figure 2:**
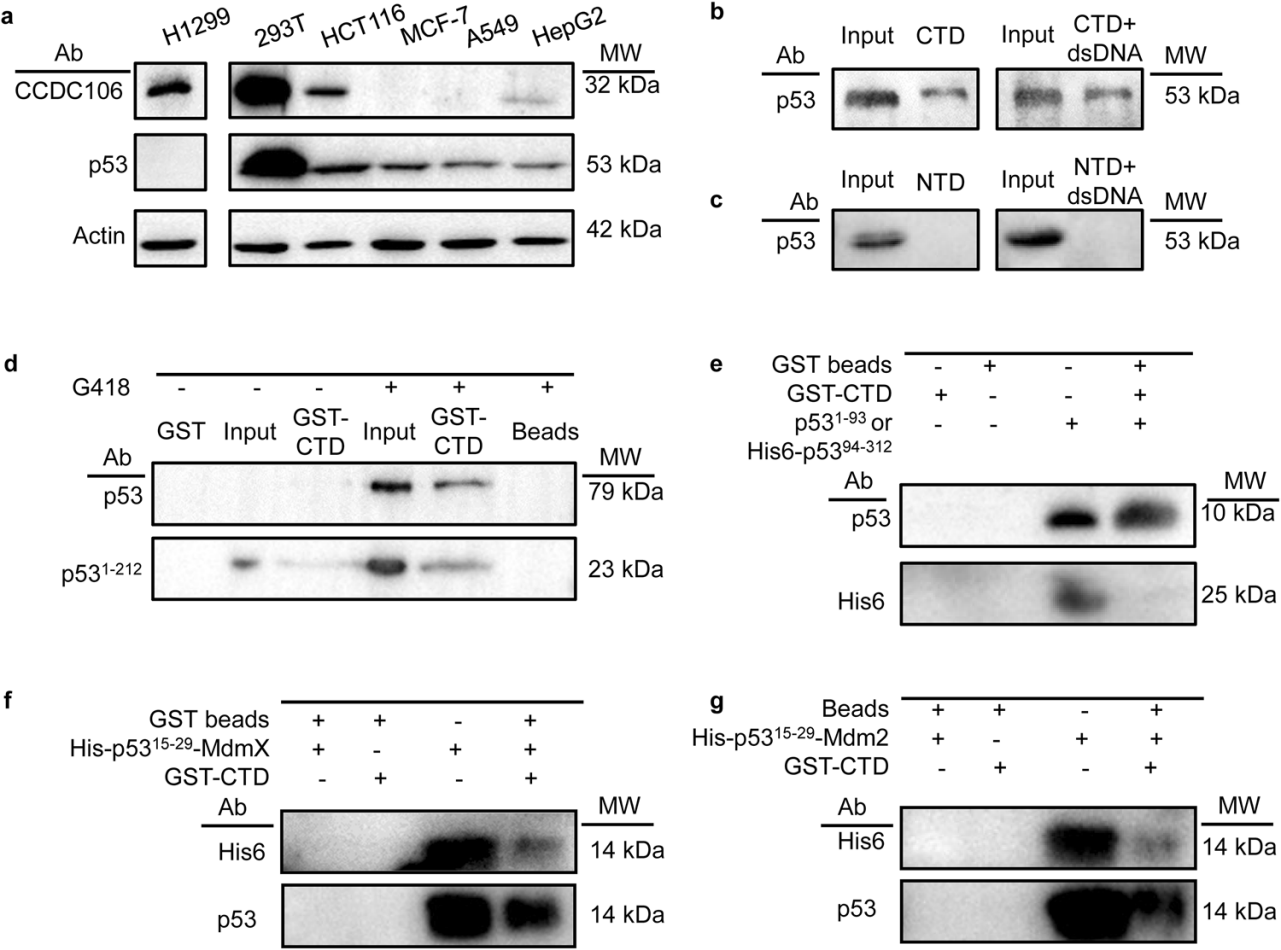
Correlation between CCDC106 and p53. GST: GST protein; Input: cell extracts; GST-CTD: GST-CTD fusion protein; Beads: GST agarose beads; p53: full-length p53 protein; p53^1-212^: N-terminal segment of p53 containing TAD, PRD and partial DBD domain; p53^1-93^: TAD and PRD domains; p53^94-312^: DBD domain of p53; p53^15-29^: p53 TAD domain; N-MdmX and N-Mdm2: N-terminal domains of MdmX and Mdm2, respectively. (**a**) The cellular levels of endogenous CCDC106 and p53 proteins in H1299, 293T, HCT116, MCF-7, A549 and HepG2 cells were assayed by western blotting. (**b**) In vitro pulldown of p53 protein with the GST-CTD fusion protein was detected with anti-p53 polyclonal antibody. (**c**) No obvious interaction was detected in vitro between p53 and GST-NTD with a GST pulldown assay that was detected with an anti-p53 polyclonal antibody. (**d**) Pulldown of the full-length and an N-terminal segment of p53 with the GST-CTD fusion protein. (**e**) Pulldown of p53^1-93^ but not p53^94-312^ with the GST-CTD fusion protein. Inputs were the cell extracts of *E. coli* cells expressing p53^1-93^ or p53^94-312^. (**f**, **g**) Pulldown of p53^15-29^ in the N-MdmX or N-Mdm2 fusion forms with the GST-CTD fusion protein. Blots in each subgroup were made from either the same gel or different gels with similar exposure times. Source data for (**a − g**) are provided as a Source Data file.

As shown in Fig. 2a, CCDC106 was overexpressed in H1299, 293T and HCT116 cells, while CCDC106 was marginally expressed in HepG2 cells. However, no significant CCDC106 protein was detected in MCF-7 and A549 cells. We noted that both p53 and CCDC106 were overexpressed in 293T and HCT116 cells, while p53 was expressed at low levels in MCF-7, A549 and HepG2 cells. We also noted that p53 is significantly overexpressed in 293T cells due to the expression of the large T antigen of simian virus 40 (SV40), which stabilizes and inactivates the p53 protein^9^. Nevertheless, in three tested cell lines that expressed CCDC106, the expression of cellular p53 protein was correlated with the cellular level of CCDC106, suggesting that the high cellular level of CCDC106 was associated with the high level of cellular p53 protein.

To examine how CCDC106 interacts with the p53 protein, we conducted in vitro GST pulldown assays using GST-NTD and GST-CTD fusion proteins. As shown in Fig. 2b, in the presence or absence of double-strand DNAs for the p53 protein, the CTD domain of CCD106 interacted with the p53 protein, while the NTD domain of CCDC106 had no interaction with the p53 protein (Fig. 2c).

### CCDC106 interacts with the p53 TAD domain

Next, we employed p53-null H1299 cells to investigate how CCDC106 interacts with the p53-Mdm2/MdmX signaling pathway in vivo. In this work, we first ensured that the full-length p53 gene was null in our H1299 cells (Supplementary Figs. S2, S3, and Supplementary Data S1–S3), as the current literature has not provided detailed information on its default p53 gene^10,11^. In engineered H1299^p53+^ cells that can express the full-length wild-type p53 protein and a truncated form of p53 protein (p53^1-212^), we used the GST-CTD fusion protein as bait to conduct a GST pull-down assay. As indicated in Fig. 2d, both full-length p53 and p53^1-212^ were pulled down from H1299^p53+^ cells, indicating that the CCDC106 binding site on p53 is located within the N-terminal region of p53. To narrow down the CCDC106 binding site on the p53 protein, we distinguished whether CCDC106 interacts with the p53-TAD/PRD domain (i.e., p53^1-93^) in comparison with the p53-DBD (i.e., p53^94-312^). As shown in Fig. 2e, the GST pulldown assay indicated that the CTD domain of CCDC106 interacted with the p53-TAD/PRD region but not the p53 DBD domain, suggesting that the CTD domain interacts with the p53^1-93^ segment on p53. To determine whether we could further narrow the binding site of CCDC106 on the p53 protein, we performed a GST pull-down assay with the p53-TAD domain (p53^15-29^) fused with the N-terminal domain of MdmX (N-MdmX). As shown in Fig. 2f, the GST-CTD fusion protein could pull down the His6-p53^15-29^-MdmX fusion protein in vitro, as detected by anti-His6 and anti-p53 antibodies. A similar interaction between GST-CTD and His6-p53^15-29^-Mdm2 was also observed (Fig. 2g). To exclude the interaction between CCDC106-CTD and N-MdmX or N-Mdm2, we used the GST-N-MdmX fusion protein to pull down a His6-tagged CTD protein (i.e., His6-CTD), as shown in Supplementary Fig. S4. The CTD domain of CCDC106 had no interaction with N-MdmX. Finally, we quantitively determined the binding affinity of p53^15-29^ for the CTD domain with a *K*_*d*_ value of 0.32 µM (Supplementary Fig. S5).

### CCDC106 downregulates p53 and Mdm2

**Figure 3:**
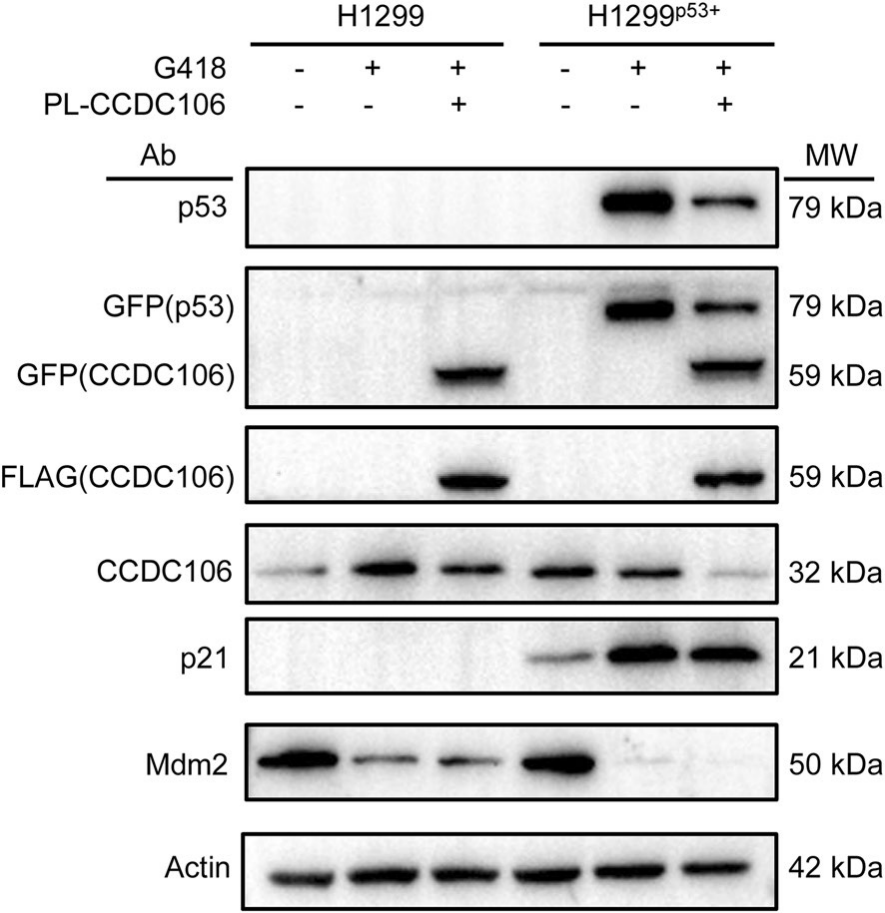
CCDC106 downregulates cellular p53 and Mdm2 in H1299^p53+^ cells. Assays were performed with western blotting in the presence or absence of G418. G418: inducer of p53 expression in H1299^p53+^ cells; PL (CCDC106): the CCDC106 expression plasmid; GFP (CCDC106) and FLAG (CCDC106): CCDC106 fused with FLAG and GFP tags; p53 protein was detected with p53 polyclonal antibody and anti-GFP tag monoclonal antibody. CCDC106 was detected with a CCDC106 polyclonal antibody, an anti-GFP tag monoclonal antibody and an anti-FLAG monoclonal antibody. p21 and Mdm2 were detected with their polyclonal antibodies. Actin was used as a control. All blots were made from the same gel. Source data are provided as a Source Data file.

It is well known that the p53-TAD domain interacts with Mdm2 and MdmX. To question whether and how the CCDC106 CTD competes with Mdmd2/MdmX to interact with p53, we used p53-null H1299 cells as a model. In H1299^p53+^ cells in which the wild-type p53 gene is fused with the GFP gene, G418 induces overexpression of the cellular p53-GFP fusion protein, which is detectable with anti-p53 and anti-GFP antibodies (Fig. 3). As shown in Fig. 3, the cellular level of the p53-GFP fusion protein is downregulated with the expression of exogenous CCDC106 protein that is detected by anti-GFP and anti-FLAG antibodies. Notably, in H1299^p53+^ cells, the cellular level of the exogenous CCDC106 protein was not significantly different from that in p53-null H1299 cells, indicating that CCDC106 overexpression promotes p53 degradation. We noted that the current concentration of G418 upregulates endogenous CCDC106 in H1299 cells, although the mechanism is not clear. However, in H1299^p53+^ cells, endogenous CCDC106 is significantly downregulated, suggesting that the overexpression of p53 downregulates endogenous CCDC106.

We also noted that the endogenous CCDC106 level significantly decreased with the overexpression of exogenous CCDC106 in comparison with that in p53-null H1299 cells and H1299^p53+^ cells that contained no CCDC106 expression plasmids (Fig. 3). It was possible that the overexpression of the exogeneous CCDC106 significantly downregulated the cellular p53 protein, causing a downregulation of the endogenous CCDC106. In contrast, the overexpression of exogeneous CCDC106 had no effect on endogenous CCDC106 in the absence of p53. Nevertheless, the cellular level of p21 protein remained at an upregulated level independent of the cellular level of CCDC106 protein. In this study, we found that H1299 cells are typical Mdm2-overexpressing cancer cell lines (Fig. 3). We also noticed that the current concentration of G418 downregulated the cellular level of Mdm2, although the mechanism was unclear. Nevertheless, this biological effect was independent of CCDC106 expression (Fig. 3). Interestingly, when both p53 and exogenous CCDC106 were overexpressed, the endogenous Mdm2 protein was significantly downregulated, accompanied by the downregulation of endogenous CCDC106 (Fig. 3). Thus, it is likely that CCDC106 regulates the level of Mdm2 via p53.

### Overexpression of Mdm2 and MdmX downregulates p53 and CCDC106

**Figure 4:**
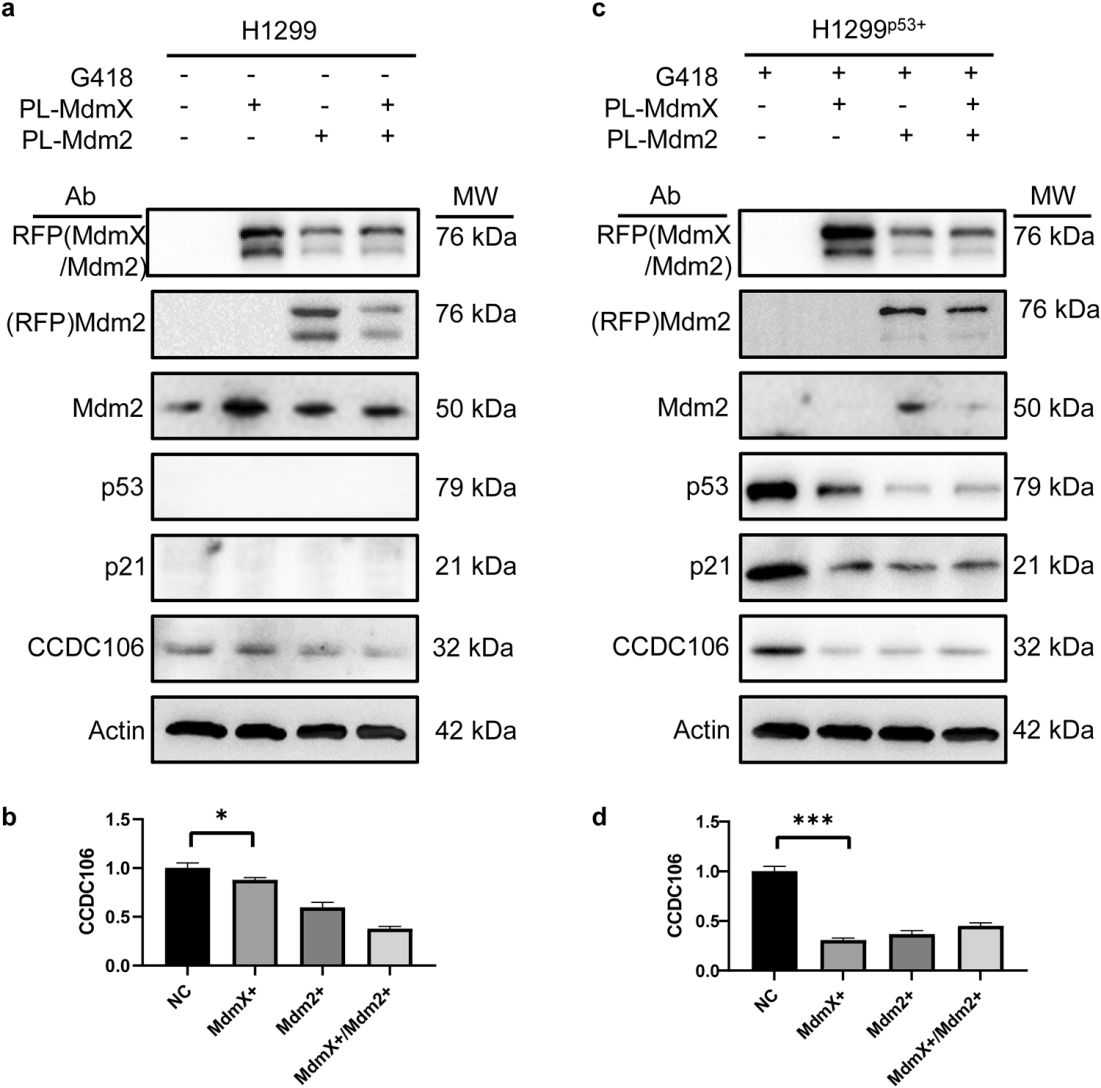
Mdm2 and MdmX downregulate cellular CCDC106 via p53. PL-MdmX and PL-Mdm2: plasmids harboring MdmX or Mdm2 genes, respectively; RFP(MdmX/Mdm2): MdmX-RFP and Mdm2-RFP fusion proteins, respectively. Exogenous MdmX and Mdm2 were detected with their polyclonal antibodies. Endogenous Mdm2 was detected with a polyclonal antibody. The p53 protein was detected with a p53 polyclonal antibody. Endogenous p21 and CCDC106 proteins were detected with p21 and CCDC106 polyclonal antibodies, respectively. Actin was used as a control. Blots in (**a**) and (**c**) were made from two different gels. Source data for (**a–b**) are provided as a Source Data file. (**b**) and (**d**) Quantitation of the expression of CCDC106 regulated by overexpressing MdmX and Mdm2. n = 3 independent experiments, *p < 0.05; **p < 0.01; ***p < 0.005, compared with the control group.

In H1299 cells, the expression levels of the exogenous MdmX and Mdm2 genes were distinctly different. The MdmX level was much higher than that of the Mdm2 protein (Fig. 4a, the left first row). Exogenous Mdm2 was also detectable (Fig. 4a, the left second row). When the exogenous MdmX and Mdm2 genes were expressed, the exogenous MdmX protein was further downregulated. Nevertheless, the expression of the exogenous MdmX or Mdm2 genes in H1299 cells exhibited no significant effects on the levels of the endogenous Mdm2 protein (Fig. 4a, the third row). Notably, in H1299 cells, the overexpression of exogenous MdmX exhibited no effect on the cellular level of endogenous CCDC106 (Fig. 4a, the sixth row), while the expression of exogenous Mdm2 slightly downregulated the cellular level of endogenous CCDC106. When both exogenous MdmX and Mdm2 were expressed, the cellular level of endogenous CCDC106 was significantly downregulated (Fig. 4a). The downregulation of CCDC106 was quantitated as a function of the expression of Mdm2 and MdmX (Fig. 4b), indicating that the co-overexpression of Mdm2/MdmX has a synergic effect on the downregulation of CCDC106 in the absence of p53.

By comparison, in H1299^p53+^ cells, the overexpression of the full-length p53 protein induced by G418 resulted in the upregulation of cellular p21 and CCDC106 levels (Fig. 4c). The overexpression of the exogenous MdmX gene significantly downregulated the levels of cellular p53, p21 and CCDC106 proteins, accompanied by Mdm2 downregulation. The overexpression of the exogenous Mdm2 gene could maintain a high cellular level of the endogenous Mdm2 protein, as detected by antibodies against RFP (Fig. 4c, the first row) and Mdm2 (Fig. 4c, the second row). The overexpression of the exogenous Mdm2 gene further downregulated the level of cellular p53 protein, causing a low level of cellular p21 protein, while the cellular level of the CCDC106 protein was maintained at a low level independent of the expression of exogenous and endogenous Mdm2 (Fig. 4c). On the other hand, the coexpression of exogenous MdmX and Mdm2 significantly downregulated the cellular level of endogenous Mdm2, while the cellular levels of the p53, p21 and CCDC106 proteins were maintained at low levels. The downregulation of CCDC106 was quantitated as a function of the expression of Mdm2 and MdmX (Fig. 4d), indicating that the co-overexpression of Mdm2/MdmX has a significant effect on the downregulation of CCDC106 in the presence of p53.

Therefore, it is likely that the overexpression of either MdmX or Mdm2 enables the downregulation of the cellular level of CCDC106 mediated only by p53. The overexpression of exogenous Mdm2 significantly downregulates the cellular level of p53 more than exogenous MdmX expression. The presence of the exogenous MdmX protein significantly reduced the cellular level of endogenous Mdm2, and this regulation was also mediated by p53. Taken together, Mdm2 and MdmX play important and different roles in regulating the cellular level of the CCDC106 protein.

### CCDC106 attenuates p53 inhibition of H1299^p53+^ cell growth

**Figure 5:**
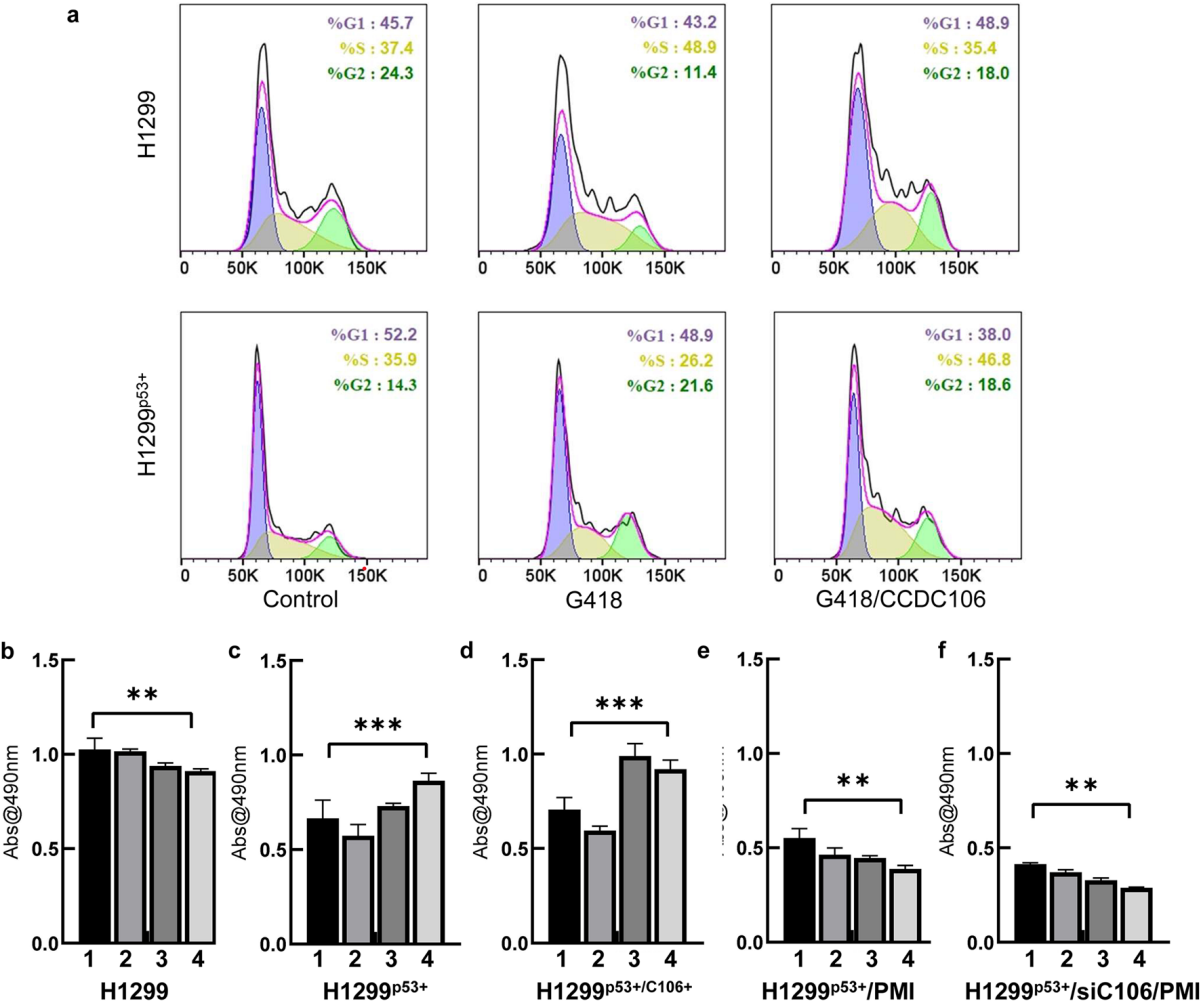
Overexpression of CCDC106 attenuates the effect of p53 on cell proliferation. All cells were cultured in the presence of 50 μg/mL G418 to induce p53 overexpression. (**a**) A flow cytometric assay of H1299^p53+^ cells overexpressing CCDC106 in comparison with H1299 cells. Control: no G418 and PL-CCDC106 plasmid. (**b–f**) Effects of the Mdm2/MdmX dual inhibitors (PMI) and CCDC106 siRNA on H1299^p53+^ cell viability in comparison with those of H1299 and H1299^p53+^ cells. 1: negative control; 2: transiently transfected with the MdmX expression vector; 3: transiently transfected with the Mdm2 expression vector; 4: transiently transfected with both MdmX and Mdm2 expression vectors. n = 3 independent experiments, *p < 0.05; **p < 0.01; ***p < 0.005, compared with the control group. (**b**) H1299 cells. (**c**) H1299^p53+^ cells. (**d**) H1299^p53+^ cells were transfected with the CCDC106 expression plasmid. (**e**) H1299^p53+^ cells were treated with 1 µM of PMI. (**f**) H1299^p53+^ cells were treated with CCDC106 siRNA and 1 µM PMI. Source data for (**b − f**) are provided as a Source Data file.

To explore how CCDC106 affects the expression of the p53 gene and thereby controls cell cycle arrest, we treated H1299^p53+^ cells and their negative control cells (i.e., H1299 cells) with G418 for 48 h in the presence or absence of the CCDC106 expression vector. As shown in Fig. 5a, our flow cytometric assay indicated that the overexpression of p53 in H1299^p53+^ cells significantly affected the cell cycle, causing cell arrest at the G2 stage (bottom middle panel), while the H1299 cell cycle arrested at the S stage (*top middle panel*). G418 is an aminoglycoside antibiotic that may inhibit protein synthesis in H1299 cells. Thus, the presence of G418 caused S-phase arrest. However, this arrest was attenuated by the expression of p53. When both p53 and CCDC106 were overexpressed in H1299^p53+^ cells, the cell numbers in the G1 and G2 stages were significantly reduced (bottom right panel) compared with those in H1299 cells (top right panel), indicating that CCDC106 overexpression attenuated p53 function in regulating the cell cycle.

Next, we evaluated the viability of H1299 and H1299^p53+^ cells in the presence of CCDC106 siRNA and the Mdm2/MdmX inhibitor by MTT experiments. As shown in Fig. 5b, an insignificant decrease in H1299 cell viability was observed when the cells were transfected with the MdmX and Mdm2 expression vectors. However, the viability of the H1299^p53+^ cells dropped much faster than that of H1299 cells when p53 was overexpressed (Fig. 5c). When the MdmX expression vector was transfected into H1299^p53+^ cells, cell viability further decreased, while H1299^p53+^ cells transfected with the Mdm2 expression vector started to recover cell viability (Fig. 5c). The transfection of the MdmX and Mdm2 expression vectors significantly recovered cell viability. Furthermore, we examined the effect of CCDC106 overexpression on the viability of H1299^p53+^ cells in the presence of overexpressed p53 proteins. As shown in Fig. 5d, the transfection of the CCDC106 expression vector with or without the transfection of the MdmX expression vector exhibited no significant effect on cell viability. However, transfection of the CCDC106 expression vector with the Mdm2 expression vector significantly recovered cell viability (Fig. 5d), comparable to that of H1299 cells (Fig. 5b). A similar enhancement was also observed for the H1299^p53+^ cells that were transfected with both the Mdm2 and MdmX expression vectors (Fig. 5d).

Thus, we considered examining how the inhibition of the cellular Mdm2/MdmX and CCDC106 proteins affects cell viability. As shown in Fig. 5e, the use of the Mdm2/MdmX dual peptide inhibitor PMI^12^ significantly attenuated the effects caused by the overexpression of MdmX and Mdm2, resulting in lower cell viability. Importantly, cell viability could be further reduced when these cells were treated with CCDC106 siRNA in combination with PMI (Fig. 5f).

## Discussion

To date, many studies have found that CCDC106 interacts with p53, resulting in p53 degradation in cancer cells and promoting cell proliferation^1,3,4,13^. In this work, we provide evidence that the CTD of the CCDC106 protein (CCDC106-CTD) directly interacts with the N-terminal domain of the p53 protein. More precisely, the CCDC106-CTD domain interacts with the TAD of the p53 protein. We observed that such protein–protein interactions affect cell proliferation, as the cellular level of the p21 protein was downregulated (Fig. 3). Certainly, it is also important to examine how the interaction of CCDC106 with p53 affects the p53 signaling axis. More p53 targets should be investigated upon the interaction.

**Figure 6:**
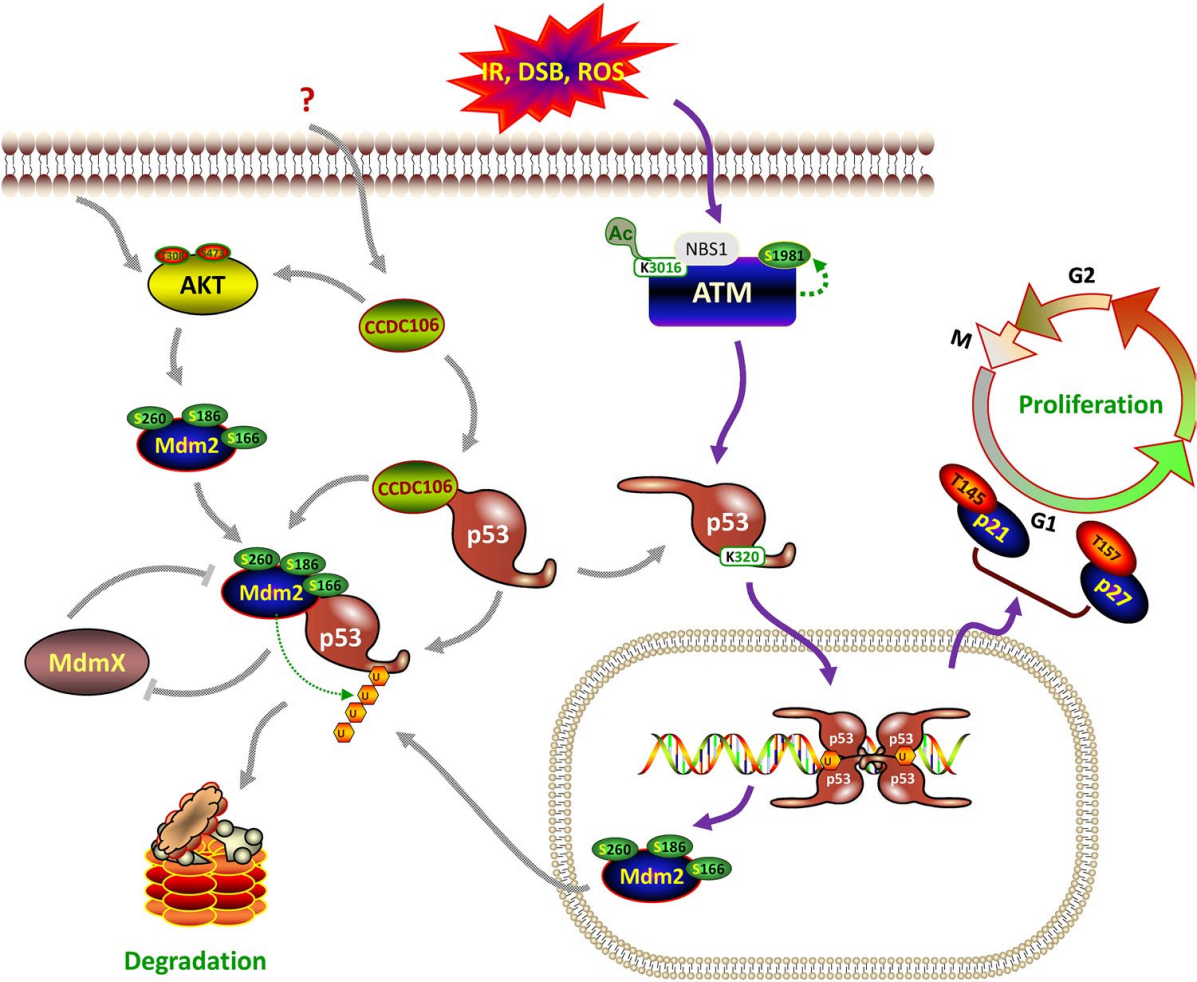
A signaling map depicting the CCDC106-p53-Mdm2 interactions regulating cell proliferation. This signaling map was constructed by combining the data from this work with previously published data^6,7^. IR: irradiation; DSB: DNA double strand break; ROS: reactive oxygen species; AKT: Akt serine/threonine kinase; ATM: ataxia telangiectasia mutated kinase; NBS1: Nijmegen breakage syndrome 1; Ac: acetyl group; T: threonine; S: serine; K: lysine; U: ubiquitin. p27: Cyclin dependent kinase inhibitor p27.

Identification of the CCDC106-p53-Mdm2/MdmX interaction immediately raises critical questions regarding how CCDC106 interferes with Mdm2/MdmX binding to p53 and how CCDC106 regulates the p53 signaling pathway, as Mdm2/MdmX are specific to the p53 TAD domain^14,15^. Despite their profound relationship to p53, Mdm2 and MdmX may have quite rich and complex lives outside of p53^16^. In this work, it was found that CCDC106 overexpression can significantly downregulate p53 and Mdm2. We also found that MdmX overexpression can downregulate Mdm2 and CCDC106. More importantly, in the absence or presence of p53, MdmX and Mdm2 exhibited different effects on the level of CCDC106. Thus, a concise interactive network between CCDC106 and the p53-Mdm2/MdmX signaling axis is summarized in Fig. 6, depicting that CCDC106 promotes p53 and Mdm2 degradation. On the other hand, we observed that the overexpression of exogenous CCDC106 increased the endogenous levels of the protein (Fig. 3).

Mdm2/MdmX are overexpressed in many cancers to impair p53 activity^15,17^, serving as a hot drug target for cancer therapy^18–21^. Considering that CCDC106 competes with Mdm2/MdmX for binding p53 identified in this work, we reason that the CCDC106-p53 interaction should be a promising drug target for cancer killing in synergetic combination with drugs targeting aberrant p53-Mdm2/MdmX interactions.

## Methods

### Cell lines and cell cultures

NCI-H1299, HCT116, MCF-7, A549, 293T and HepG2 cells were purchased from ATCC, and the NCI-H1299^p53+^ cell line was constructed in our group^22,23^. All kinds of cells were generally cultured in a 37 °C incubator with 5% CO_2_ according to ATCC protocols in RPMI-1640 medium (Gibco, USA) containing 10% fetal bovine serum (FBS, Gibco, USA) supplemented with 10% serum, penicillin and streptomycin. After the cell confluency reached 80%, an individual plasmid harboring a gene encoding Mdm2, MdmX or CCDC106 tagged with RFP protein was individually or in combination transfected to overexpress exogenous Mdm2-GFP, MdmX-GFP and FLAG-CCDC106-GFP fusion proteins.

### GST pulldown experiments

Pulldown experiments were carried out with GST-NTD or GST-CTD beads to pull down p53 in cancer cell lines. GST-agarose beads were also used to examine the interaction of CTD with different domains of the p53 protein, i.e., p53^15-29^ (the p53 binding peptide, p53p), p53^1-93^ (transactivation domain, TAD), p53^94-312^ (DNA binding domain, DBD) and full-length p53. The GST pulldown samples were subjected to western blotting.

### Western blotting assay

Total cellular proteins were extracted with RPMI lysis buffer containing 50 mM Tris (pH 7.4), 150 mM NaCl, 1% Triton X-100, 0.1% sodium deoxycholate, 0.1% SDS and 0.1% PMSF. The concentration of the protein extracts was first measured with Nanodrop-2000c at _OD280 nm_, and the cellular β-actin contents were then calibrated with mouse anti-β-actin monoclonal antibody from ProteinTech (Wuhan, China; Cat#: HRP-60008; Gene ID 60, 100 μg/ml) with a dilution ratio of 1:10,000. Calibrated samples were separated by SDS-PAGE, while a normal molecular marker was used. Each SDS-PAGE gel was electrically transferred to a polyvinylidene difluoride (PVDF) membrane (Millipore, USA). To minimize the usage of antibodies, each PVDF membrane was cut into different sections based on the molecular weights of cellular p53, Mdm2, MdmX, p21, CCDC106 and β-actin and the features of their corresponding antibodies provided by their manufacturers. Each PVDF membrane section was blocked with 5% skimmed milk powder dissolved in TBST buffer for 1 h at room temperature, followed by incubation individually with their corresponding primary antibodies for 2 h in a sealed plastic bag. After washing 3 times with TBST, the membranes were incubated with HRP-conjugated secondary antibody for 1 h in a sealed plastic bag. Multiple sections of PVDF membranes were collected, and the protein bands were visualized using enhanced chemiluminescence (ECL) reagents from BioSharp (Beijing, China) on a Tanon 5200 Chemiluminescent Imager (Shanghai, China). Each blotting was repeated until a good quality image was achieved. Cellular p53 protein was blotted with a rabbit anti-TP53 polyclonal antibody IgG from CUSABIO (TX, USA; Cat#: CSB-PA15509AORB, Lot#: F0912A) with a dilution ratio of 1:4000; cellular Mdm2 and MdmX proteins tagged with RFP were assayed with a mouse anti-RFP monoclonal antibody from Solarbio (Beijing, China; Cat#: K20016M) with a dilution ratio of 1:10,000. Cellular p21 protein was detected with a rabbit anti-p21 polyclonal antibody from Elabscience (Wuhan, China; Cat#: E-AB-40097) with a dilution ratio of 1:500. Cellular CCDC106 protein was detected with a rabbit anti-human CCDC106 polyclonal antibody from Abnova (Wuhan, China; Cat#: PAB19286; protein ID NP_037433) with a dilution ratio of 1:1000. Primary antibodies were detected with either HRP-conjugated goat anti-rabbit IgG (H + L) from ProteinTech (Wuhan, China; Cat#: SA00001-2) with a dilution ratio of 1:10,000 or HRP-conjugated goat anti-mouse IgG (H + L) from Biosharp (Guangzhou, China; Cat#: BL001A, 0.8 mg/ml) with a dilution ratio of 1:10,000.

### Flow cytometric assay

Cells were counted and plated in 6-well plates at 1 × 10^5^ cells/ml in RPMI 1640 culture medium supplemented with 10% FBS. After being incubated for 3 days, the cells were washed twice with cold PBS buffer and resuspended in 1 × PBS buffer at 1 × 10^6^ cells/mL. The cell samples were incubated with Alexa Fluor488 Annexin V and PI work solution (Dead Cell Apoptosis Kit) at room temperature for 15 min. The cell cycles were analyzed by propidium iodide (PI) reagent with a BD FACSMelody flow cytometer.

### Assessment of cell viability

MTT assays were performed on a Synergy H1 multiplate reader (Biotek, USA) using H1299 cells and H1299^p53+^ cells. Cells were grown at 37°C with 5% CO_2_ in RPMI 1640 culture medium supplemented with 10% FBS until the cell density reached 90% confluency. Cells were counted and plated in 96-well plates at 5 × 10^3^ cells per well. After incubation for 3 days, the medium was replaced with RPMI 1640 supplemented with 3% FBS. G418 was added at a final concentration of 50 μg/ml, and the cells were cultured for 48 h before PMI was added at a final concentration of 1 μM in the absence or presence of siRNAs.

Then, MTT (5 mg/ml) solution was added to 10 μl/well. The cells were incubated for another 4 h at 37°C. After discarding the culture media, the cells were washed with PBS, followed by the addition of 100 μl DMSO to each well. The plates were shaken on a plate shaker for 10 min. The plates were then read with the plate reader at OD_490 nm_. Data were processed with MicroCal Origin software (v2017, MicroCal, USA).

### Statistical analysis

The data are expressed as the mean ± standard error (SEM), and statistical analysis was performed using GraphPad Prism 5 Software. Differences between two independent samples were analyzed using Student's t-test. A *p* value of less than 0.05 was considered significant.

### Supplementary Information

Supplementary Information 1.

Supplementary Information 2.

## Data Availability

The nucleotide sequence of the p53 genome in the H1299 cell line generated in this study has been deposited in GenBank under Submission #2667019 (will be updated upon available).
