# Supplementary material for: Molecular mechanism of CCDC106 regulating the p53-Mdm2/MdmX signaling axis
Source: Sci Rep. 2023 Dec 11;13:21892. doi: 10.1038/s41598-023-47808-z (PMC10713525; doi:10.1038/s41598-023-47808-z)
Supplement: Supplementary file 1 — Supplementary Information 1. [file 41598_2023_47808_MOESM1_ESM.pdf]

Supplementary Information for

Molecular mechanism of CCDC106 regulating the p53-Mdm2/MdmX signal axis

Ting Zhou[1,2,#], Zhiqiang Ke[2,3,#], Qianqian Ma[2], Jiani Xiang[2], Meng Gao[2], Yongqi Huang[2], Xiyao Cheng[1,2,] and Zhengding Su[2,*]*

[1]School of Light Industry and Food Engineering, Guangxi University, No. 100, Daxuedong Road, Xixiangtang District, Nanning, Guangxi, 530004, China.

[2]Protein Engineering and Biopharmaceutical Sciences Group, Hubei University of Technology, Wuhan 430068, China.

[3]Hubei Key Laboratory of Diabetes and Angiopathy, Xianning Medical College, Hubei University of Science and Technology, 437100 Xianning, Hubei, China

# These authors contributed to this work equally.

To whom correspondence should be addressed: Zhengding Su, Email: zhengdingsu@hbut.edu.cn, Tel.: 86-156-23901978, ORCID: 0000-0003-3558-001X or Xiyao Cheng, Email: xiyaocheng@gxu.edu.cn, Tel: 86-190-17095527, ORCID: 0000-0003-2161-1743

Keywords: CCDC106; p53; Mdm2; MdmX; p21; cell cycle; apoptosis; cancer, NSCLC.

Supplementary Results

Bioinformatic characterization of CCDC106 protein

As its biological function has not been thoroughly investigated, we use the Simple Modular Architecture Research Tool (SMART) [1] to predict its putative domains. As shown in **Fig. 1b**, it contains a coiled coil domain predicted with confidence (**Supplementary Table 1**). However, it is also likely that CCDC106 may have potential to constitute other functional domains, such as Basic region leucin zipper (BRLZ), Parathyroid hormone (PTH), Repeats in fly CG4713, worm Y37H9A.3 and human FLJ20241 (DM14), Suppressor of glucose by autophagy (SOGA), Homeobox associated leucin zipper (HALZ), Worm-specific N-terminal domain (WSN), Helicase and RNaseD C-terminal (HRDC) domains, although their e-values are over threshed (**Supplementary Table 2**).

Supplementary Table 1. Confidently predicted of domains, repeats, motifs and features of CCDC106

Structure property	Sequence start	Sequence end	E-value
Low complexity	45	57	N/A
Coiled coil	63	101	3.9e-100
Low complexity	133	150	N/A

Supplementary Table 2. Possible structure features of CCDC106

Name	Start	End	E-value	Reason
SOGA	58	157	0.21	threshold
BRLZ	43	95	32.2	threshold
PTH	49	85	60.1	threshold
HALZ	241	278	583	threshold
WSN	187	249	683	threshold
HRDC	186	265	893	threshold
DM14	55	114	1140	threshold

As predicted using alphaFold2 program, the CCDC106 structure contains a long α-helix flanked by two coils at the two ends of the α-helix in the N-terminal region of the CCDC106 protein, while its C-terminal region forms a compact helix-rich structure. Therefore, we arbitrarily define these two regions as the N-terminal domain (NTD) and the C-terminal domain (CTD) of CCDC106, respectively (**Fig. S1**).

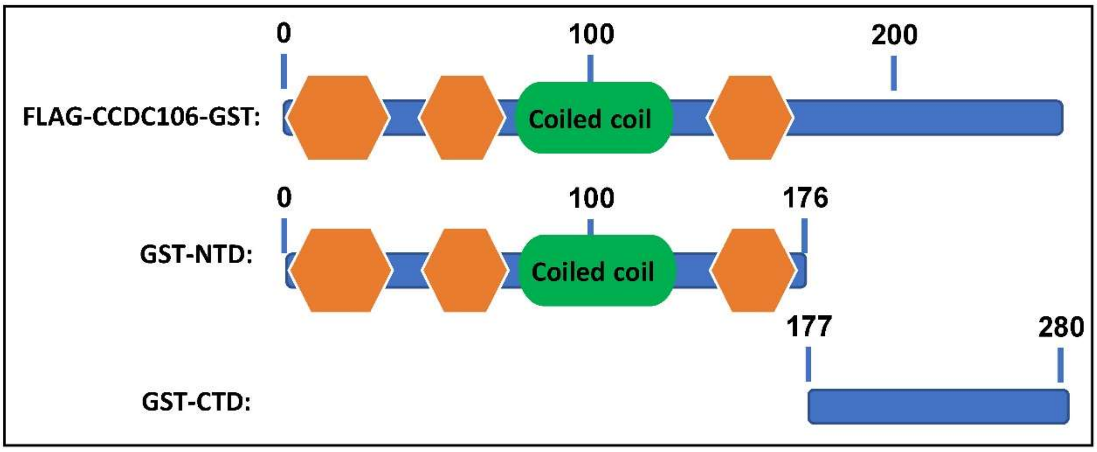

Supplementary Fig. 1. Dissection of CCDC106 structure for expression in GST fusion protein.

Examination of p53 gene in H1299 genome

In normal human cells, the loco of the wild type p53 gene is in the 17th chromosome with a length of 21070 bp embedded in a long promotor region and the p53 genome. The p53 genome contains 11 exons and 10 introns (**Supplementary Fig. 2**). We used 18 pairs of specific primers to amplifyp53 DNA fragments from the H1299 genome using the p53 genome of the 293T cells as template. As shown in **Supplementary Fig. 2b**, the fragments 11, 12 and13 were not detected from the H1299 genome, while these three fragments could be amplified from the HCT116 genome (**Supplementary Fig. 2c**) that contains a wild type p53 gene [2]. Thus, the p53 genomic DNA in H1299 cells was missing the entire DNA sequence covering the region from exon 2 to exon 7

(**Supplementary Fig. 2d** & **2e**). After amplified DNA fragments were evaluated by DNA sequencing, we found that all the amplified fragments matched the DNA sequences expected from human genomics and the p53 genome of HCT116 cells (see **Supplementary Data 1**).

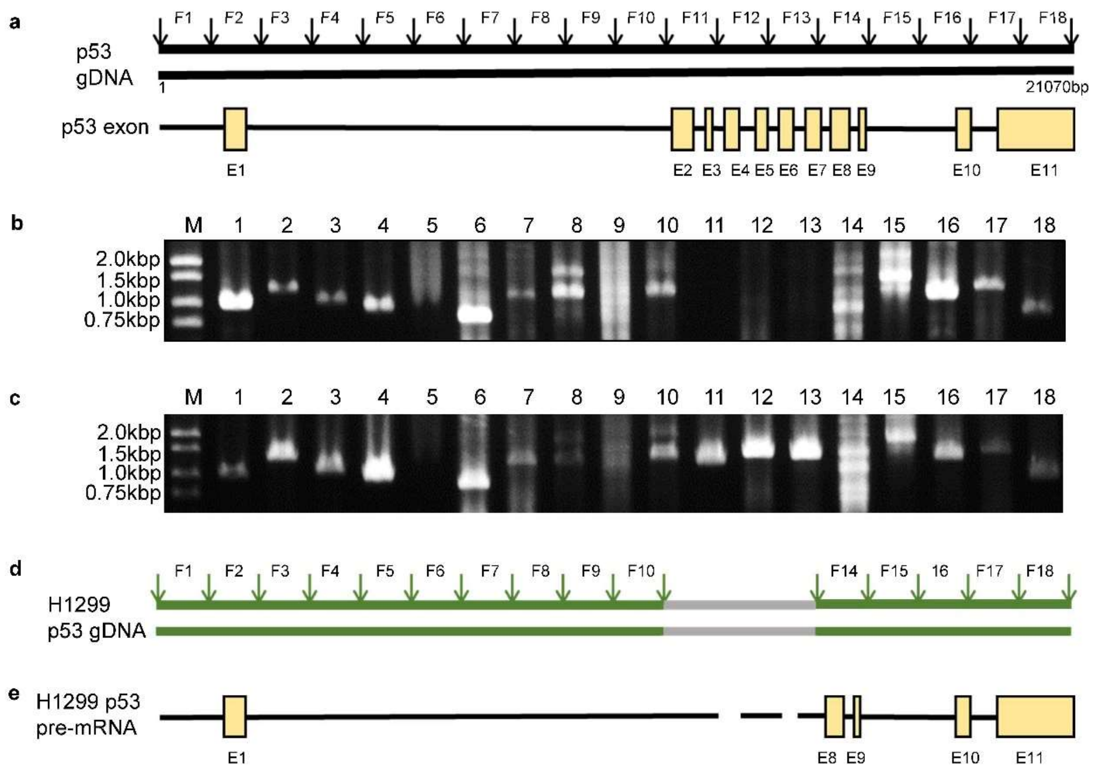

Supplementary Fig. 2. DNA sequencing of the p53 gene in H1299 cell line. a. A schematic structure of p53 genome from human genomics. F1-F18 represent 18 fragments for designing primers for sequencing the p53 gDNA. E1-E11 represent 11 annotated exons in native p53 gDNA. **b**. Agarose gel imaging of the PCR fragments for F1-F18 using the H1299 gDNA as template. **c**. Agarose gel imaging of the PCR fragments for F1-F18 using the HCT116 gDNA as template (positive control). **d**. A cartoon summarizes DNA sequencing results. *Green*: detected segments and *grey*: undetected segments in the p53 gDNA from the H1299 genome. **e**. A cartoon represents the primary mRNA of p53 in H1299 cells. Boxes represent existed exons.

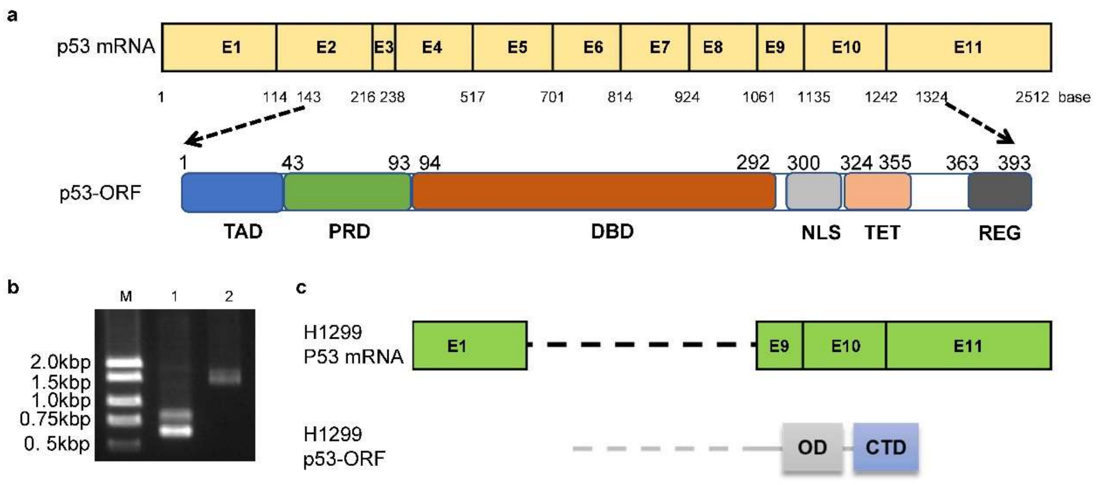

Supplementary Fig. 3. Determination of p53 mRNA in H1299 cells. a. The wild type p53 mRNA from human genomics is composed of 11 exons. An open reading frame (ORF) of matured p53 protein is deduced based on the p53 mRNA. TAD: Transactivation domain; PRD: Proline-rich domain; DBD: DNA binding domain; NLS: Nuclear localization sequence; TET: Tetramerization domain; REG: Regulation domain. **b**. The p53 cDNA reversely-transcripted from the H1299 mRNA sample was visualized by agarose gel.

The wild type p53 gene is composed of exons 2-11 (**Supplementary Fig. 3a**). And a matured p53 mRNA encodes a full-length polypeptide of 393 residues, consisting of multiple functional domains including a transactivation domain (TAD), an proline-rich domain (PRD), DNA-binding domain (DBD), nuclear localization sequence (NLS), Tetramerization domain (TET) and C-terminal regulatory domain (REG) [3] (**Supplementary Fig. 3a**). To examine whether an p53 fragment exist in H1299 cells, we isolated the total RNAs from the H1299 cell extracts and amplified p53 cDNA with two pairs of primers designed based on native p53 mRNA sequence. One pair of primers was used to amplify the region from promotor to stop codon and other pair was used to

amplify whole region from promotor to terminator. As shown in **Supplementary Fig. 3b**, the resultant PCR products was shorter than expected length. These PCR bands were further subcloned with a TOPO-Blunt vector for DNA sequencing. The DNA sequencing results revealed a shorter p53 mRNA that contains only exons 1, 9, 10 and 11 (**Supplementary Fig. 3c** and **Supplementary data 1-3**). This short mRNA can be only translated into a short peptide containing TET and REG domains (**Supplementary Fig. 3c**). Thus, our data revealed that H1299 cells cannot express a full-length p53 protein.

No interaction between CCDC106-CTD and N-MdmX

To exclude the interaction between CTD and N-MdmX or N-Mdm2, we used the GST-N-MdmX fusion protein to pulldown a His-tagged CTD protein (i.e., His6-CTD), as shown in **Supplementary Fig. 4**, the CTD domain of CCDC106 had no interaction with MdmX.

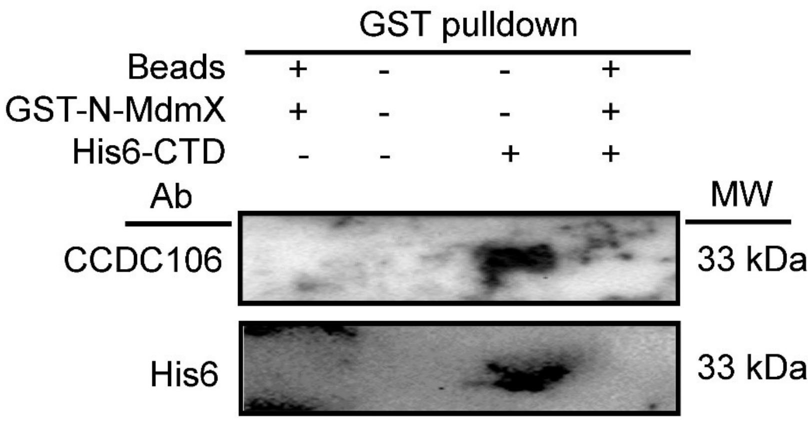

Supplementary Fig. 4. GST pulldown assay of CCDC106-CTD. Beads: GST agarose beads; GST-N-MdmX: the GST and N-MdmX fusion protein; His6-CTD: His6-tagged CTD protein. CCDC106 protein was detected CCDC106 polyclonal antibodies. Source data are provided as a Source Data file.

Quantitation of the interaction of CCDC106-CTD with p53$^{15-29}$

We quantitively determined the binding affinity of p53$^{15-29}$ for the CTD domain with a K_d value of 0.32 μM (**Supplementary Fig. 5**).

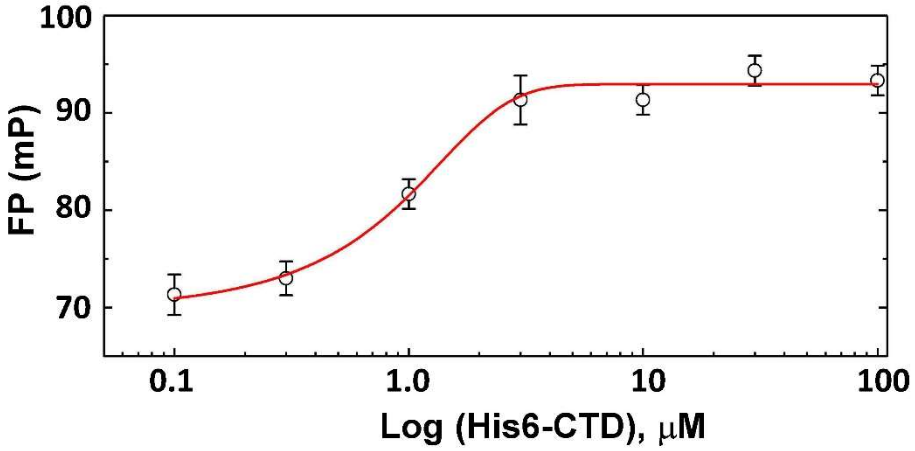

Supplementary Fig. 5. Determination of the binding affinity of p53$^{15-29}$ with the CTD domain of CCDC106 using fluorescence polarization (FP) assay. Source data are provided as a Source Data file.

Supplementary methods

Materials

The cDNA of the human CCDC106 gene was synthesized by GenScript (Wuxi, China) and sub-cloned to the pcDNA3.0 plasmid where the FLAG tag and the RFP tag were inserted at its 5-terminal and at its 3'-terminal ends, respectively. Lipofectamine 2000 was purchased from Invitrogen (Shanghai, China). The peptides including PMI and fluorescein-labeled p53p (Flu-p53p) were synthesized by TOPE Biotech (Shanghai, China). NCI-H1299, HCT116, MCF-7, A549, 293T and HepG cells were purchased

from ATCC, and NCI-H1299^{p53+} cell line was constructed in our group[4,5].

The primers used in this study were synthesized by GenScript (Nanjing, China). The siRNAs were designed and synthesized by Genepharma (Suzhou, China). Genomic DNA extraction kit, plasmid mini-prep kit and PCR cleanup kit were purchased from Tiangen Biotech (Beijing, China). RNA extraction kit RNeasy® Mini Kit was purchased from QIAGEN China (Shanghai, China). RT-PCR kits and Taq Mix were purchased from Vazyme Biotech (Nanjing, China). TOPO-Blunt Simple Cloning Kit was purchased from Yisheng Biotech (Shanghai, China). MTT (3-[4,5-dimethylthiazol-2-yl]-2,5 diphenyl tetrazolium bromide) was obtained from Biofroxx (Guangzhou, China).

Protein expression and purification from *E. coli* cells

The DNA sequences of the N-terminal domain (1-176) and C-terminal domain (177-280) of CCDC106 were optimized with *E. coli* bias codons and synthesized by GenScript (Wuxi, China) and sub-cloned to the pGEX-6P-1 plasmid. The wild-type p53 and its TAD domain (1-93) and DBD domain (94-312) were subcloned in a modified pET28b plasmid that its thrombin cleavage site was substituted with the Tev protease cleavage site[6].

Recombinant proteins were prepared in *E. coli* BL21 (*DE3*) cells. Cells were grown in LB medium containing kanamycin (34 μg/mL) for modified pET28b vector or ampicillin (50 μg/mL) for pGEX-6P-1 vector and induced with 0.4 mM IPTG at 18°C for 12 h.

To purify His-tagged protein, cells were harvested by centrifugation at 5,000 ×*g* for 30 min, resuspended in a buffer containing 10 mM Tris-HCl, 40 mM NaCl, 2 mM β-mercaptoethanol, 2 mM imidazole, pH 8.0 (Buffer A), and lysed by sonication and

homogenization, followed by spinning at 18,000 ×*g* for 30 min. The supernatant was loaded onto a 5 mL Ni-NTA agarose column (Qiagen, USA) and His-tagged protein was competitively eluted using a gradient of Buffer A mixed with Buffer B containing 10 mM Tris-HCl, 40 mM NaCl, 2 mM β-mercaptoethanol, 300 mM imidazole, pH 8.0. The eluate was diluted in 20 times with a buffer containing 20 mM sodium citrate (pH 6.5), 10% glycerol, 2 mM β-mercaptoethanol.

To purify GST-tagged protein, cells were harvested by centrifugation at 5,000 ×*g* for 30 min, resuspended in 1x PBS buffer and lysed by sonication and homogenization, followed by spinning at 18,000 ×*g* for 30 min. The supernatant was loaded onto a 5 mL GST agarose column (GE, USA) and GST-tagged protein was competitively eluted using 1x PBS buffer containing10 mM glutathione. The eluate was desalted with a 1xPBS buffer. All purified protein samples were freshly frozen in liquid nitrogen, and kept at -80 °C.

DNA sequencing of p53 genomic gene in H1299 cells

The genomic DNA of H1299 and HCT116 cells was extracted with the cell genome extraction kit, and the 53 genome was divided into 18 fragments according to the genome data on NCBI (Genebank ID: 7157). PCR was used to identify whether each fragment existed in H1299, and HCT116 was used as a control. PCR was carried out with *Taq* DNA polymerase in 50 μL reaction mixture, which contained 25 μL of 2 × Taq mix, 2 nM primers and 10 ng template DNA.

The total RNA of H1299 cells was extracted using the RNA extraction kit, and immediately reverse transcription PCR (RT-PCR) was performed to obtain the cDNA library of H1299, and then each putative fragment of p53 gene were amplified with designed primers (**Supplementary Table 3**). PCR products were evaluated by agarose

gel electrophoresis. The fragments obtained by the above PCR were purified and ligated with TOPO-Blunt vector. The ligation mixture was transformed into *E. coli* DH5α competent cells for selecting correct colonies. After cultured for 12 hours, a single colony was picked and identified using colony PCR with primers M13-F: 5'-TGTAAAACGACGGCCAGT-3' and M13-R: 5'-CAGGAAACAGCTATGACC-3'. PCP products were directly used for DNA sequencing.

Supplementary Table 3. Primers for sequencing p53 genomic DNA and cDNAs

Primer	Sequence (5′-3′)
1	TGCTCAAGACTGGCGCTA
2	AAAAAGAAATGCAGGCGGAGAATAG
3	CGATGAGAGGGGAGGAGAGAGA
4	ATATATACAACATGAACGAAT
5	GGAATCATACATTATGTG
6	CAAAGAAAAAAAGAAAATAGC
7	CCTTTCTCTACTGAATGCTTT
8	CTGGCCTATTTATCCTTTTT
9	ATGCAACAGCTAACCAATTTT
10	TAGGCCTCCCAAAGTGCTGGCAT
11	GTCGGAGTTCCACTAGCAGCA
12	GCGTGAGACATCGGGCCACTAA
13	GCCTGGGCGACAGAGCAAGACTGT
14	ATTACAGGCGCCCACCACTACA
15	TGAGACCAACCTAACATGGTG
16	TACCTAGTACTCTGTGTATTA
17	GCAGAAAGAGCTAACCTTTGTT
18	CGGAGTCTCGCCCTGTCACC
19	AATCCCAGCTACTCAGGAAGT
21	GGGCTGAGGAGTGTCCGAAGA
22	TGGGTCTTCAGTGAACCATTG
23	CTTTTCACCCATCTACAGTCC
24	GCAACCAGCCCTGTCGTCTCT
25	GCACATGACGGAGGTTGTGAG
26	CTACCTGTCCCATTTAAAAAA
27	TCCTCCACCTACCTGGAGCTG
28	GCTATGATCACATCACTGTAA
29	GCCTGCCTAGCCTACTTTTAT
30	TGAGCCAGTGCGCCTGGCCTTTT
31	AGCATGGTTGCATGAAAGGAG
32	TCAACCGGAGGAAGACTAAAA
33	CCATTCTCATCCTGCCTTCAT
34	TGGTTAGTACGGTGAAGTGGG

35	GGAGATGTAAGAAATGTTCTT
36	TGGCAGCAAAGTTTTATTGTA
p53-promoter-F	CTCAAAAGTCTAGAGCCACCG

Fluorescence polarization (FP) assay

Fluorescence polarization assay was done using black, low-protein-binding 96-well plates (Corning, NY) in a total volume of 100 μL per well of 20 mM phosphate (pH 6.8), 200 mM NaCl, and 1 mM DTT. One nM off luorescein-p53p (fluorescein-GSGSSQETFSDLWKLLPEN, Flu-p53p) titrated with GST-CTD and FP readings were taken with a 555 nm excitation filter and a 632 nm static and polarized filter on a BioTek H1 multiplate reader with Gen5 software. All FP data were fitted with Origin 2017 for obtaining K_d values.

Supplementary References

1 Letunic, I., Khedkar, S. & Bork, P. SMART: recent updates, new developments and status in 2020. *Nucleic Acids Research* **49**, D458-D460, doi:10.1093/nar/gkaa937 %J Nucleic Acids Research (2020).

2 Leroy, B. *et al.* Analysis of TP53 Mutation Status in Human Cancer Cell Lines: A Reassessment. *Human Mutation* **35**, 756-765, doi:https://doi.org/10.1002/humu.22556 (2014).

3 Zhang, S. C. L. H. B. L. S. A. A. A. U. T. X. E.-D. W. S. T. I. A. S. f. T. T. o. W.-T. & Mutant p53 in, C. *Biomolecules* **12** (2022).

4 Zhou, J. *et al.* A Protein Biosynthesis Machinery Strategy for Identifying P53PTC-Rescuing Compounds as Synergic Anti-Tumor Drugs. **3**, 11048-11053, doi:https://doi.org/10.1002/slct.201802635 (2018).

5 Cheng, X. *et al.* Premature termination codon: a tunable protein translation approach. **73**, 80-89, doi:10.2144/btn-2022-0046 (2022).

6 Qin, L. *et al.* Effect of the Flexible Regions of the Oncoprotein Mouse Double Minute X on Inhibitor Binding Affinity. *Biochemistry* **56**, 5943-5954, doi:10.1021/acs.biochem.7b00903 (2017).
