# Supplementary material for: Molecular mechanism of CCDC106 regulating the p53-Mdm2/MdmX signaling axis
Source: Sci Rep. 2023 Dec 11;13:21892. doi: 10.1038/s41598-023-47808-z (PMC10713525; doi:10.1038/s41598-023-47808-z)
Supplement: Supplementary file 2 — Supplementary Information 2. [file 41598_2023_47808_MOESM2_ESM.zip › Fig2_3_4/fig2a.pptx]

## Slide 1
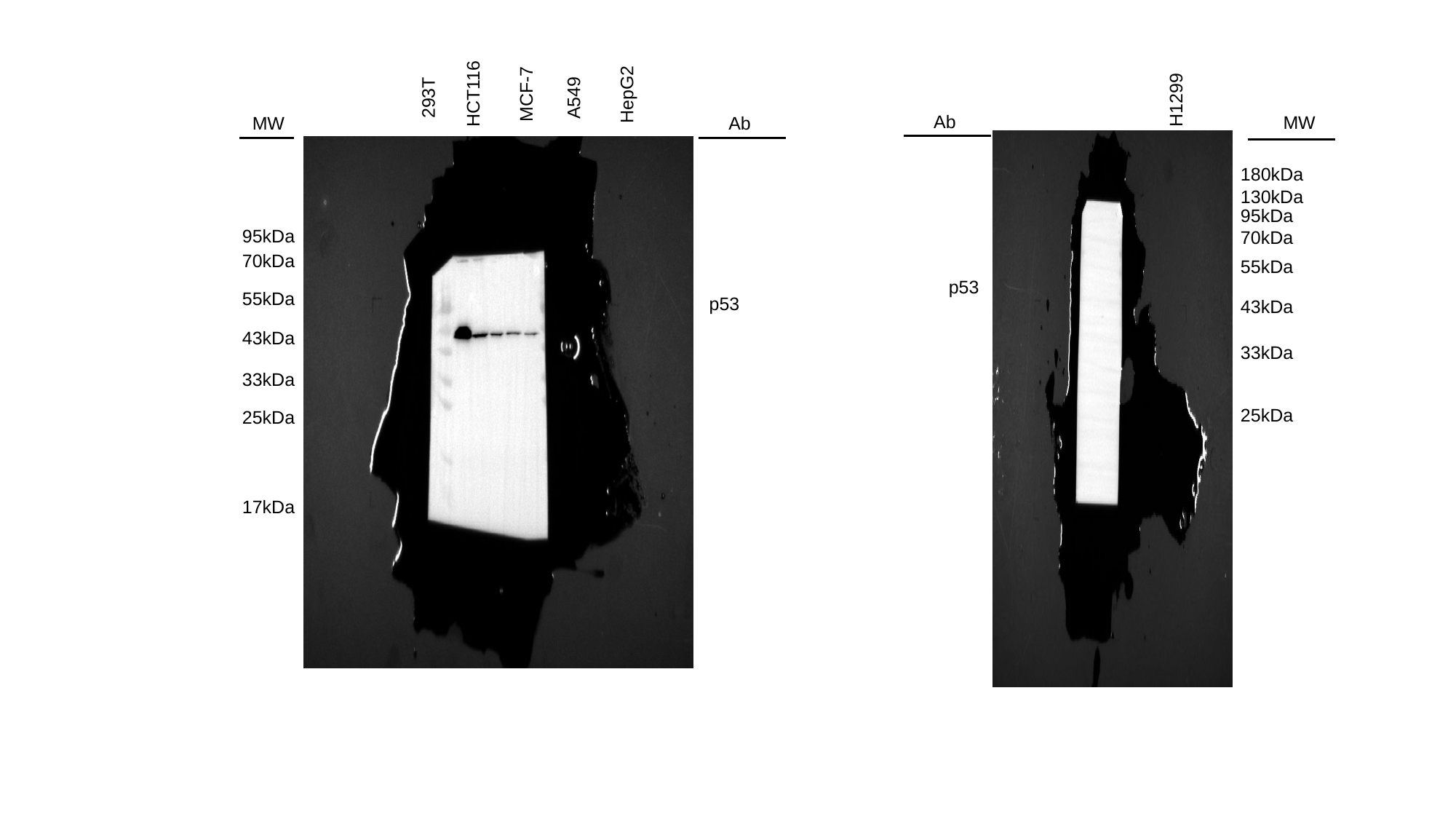

HCT116
MCF-7
HepG2
293T
A549
MW
Ab
p53
H1299
Ab
MW
180kDa
130kDa
95kDa
95kDa
70kDa
70kDa
55kDa
p53
55kDa
43kDa
43kDa
33kDa
33kDa
25kDa
25kDa
17kDa

## Slide 2
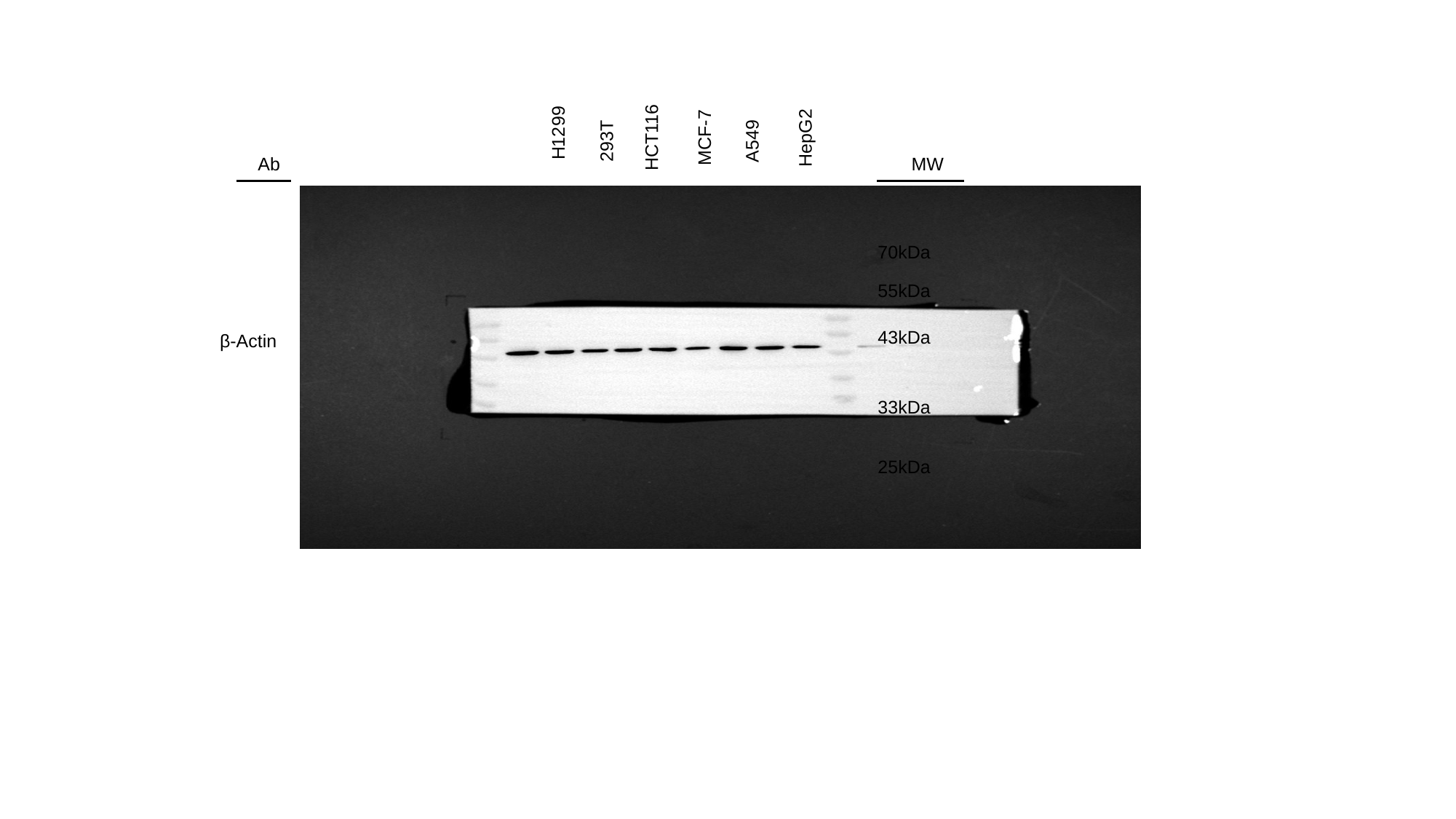

HCT116
MCF-7
HepG2
293T
A549
Ab
β-Actin
H1299
MW
70kDa
55kDa
43kDa
33kDa
25kDa

## Slide 3
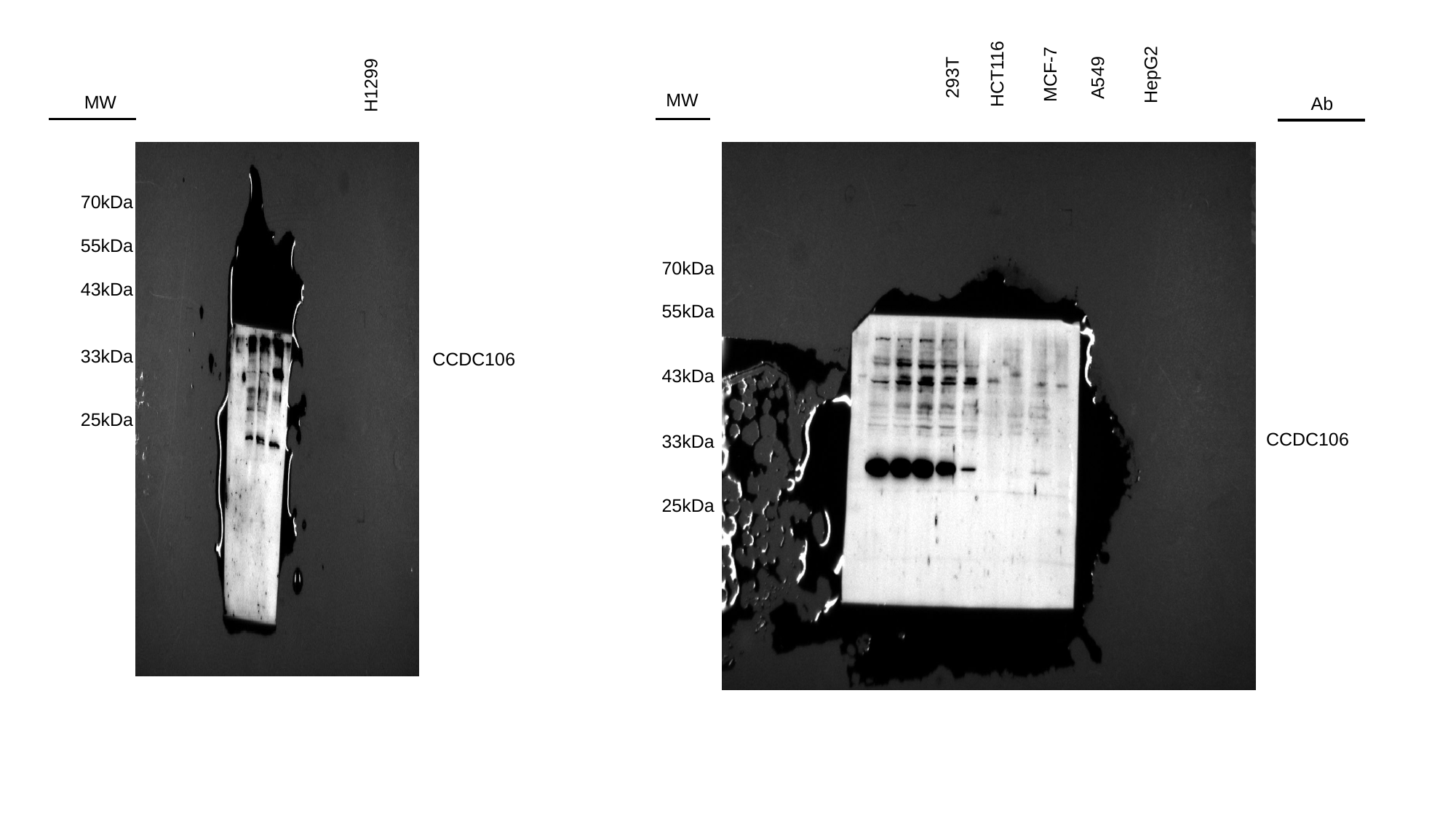

HCT116
MCF-7
HepG2
293T
A549
Ab
CCDC106
H1299
MW
MW
70kDa
55kDa
70kDa
43kDa
55kDa
33kDa
CCDC106
43kDa
25kDa
33kDa
25kDa
