# Supplementary material for: Molecular mechanism of CCDC106 regulating the p53-Mdm2/MdmX signaling axis
Source: Sci Rep. 2023 Dec 11;13:21892. doi: 10.1038/s41598-023-47808-z (PMC10713525; doi:10.1038/s41598-023-47808-z)
Supplement: Supplementary file 2 — Supplementary Information 2. [file 41598_2023_47808_MOESM2_ESM.zip › Fig2_3_4/fig2b.pptx]

## Slide 1
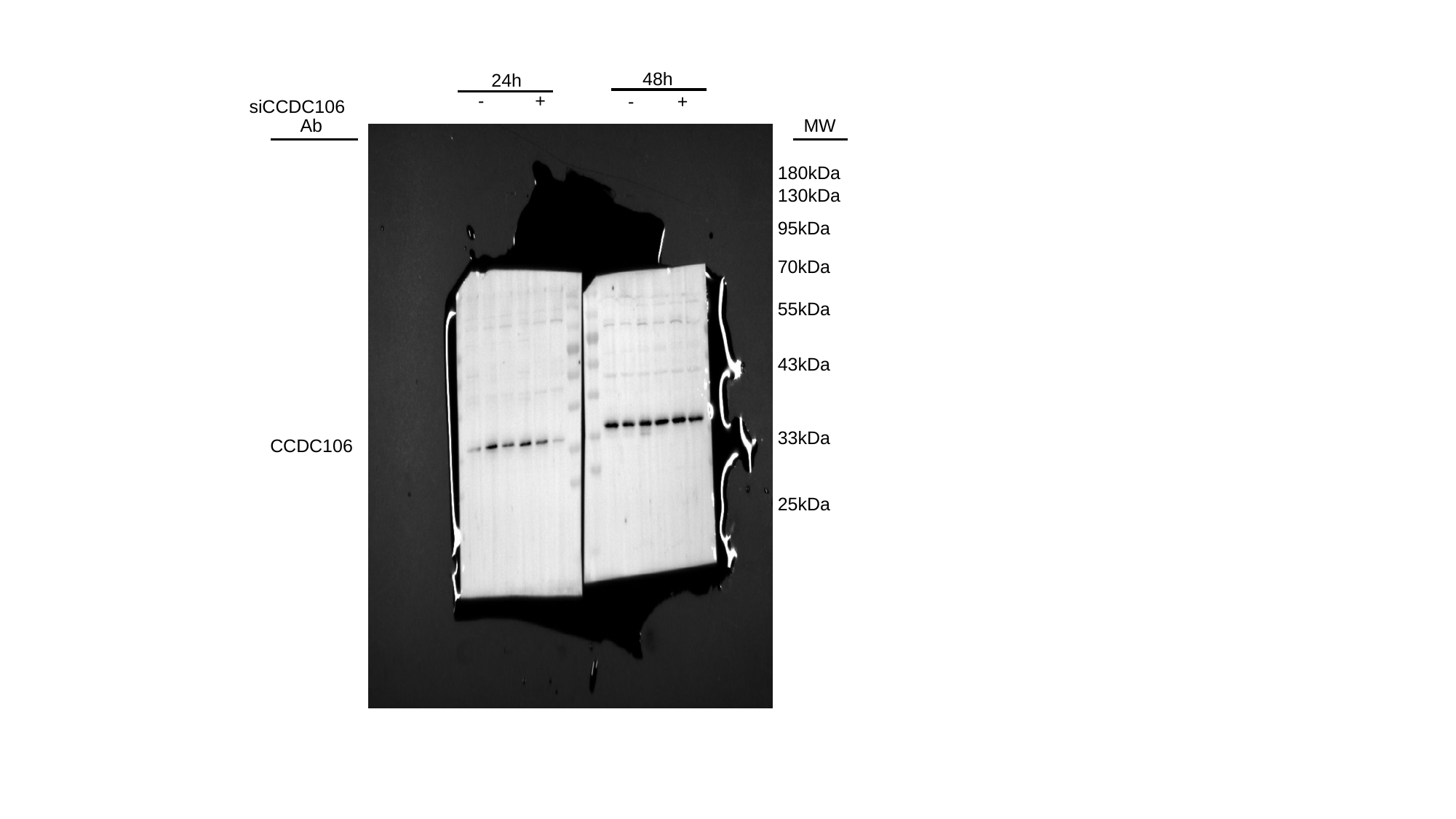

48h
24h
-
+
-
+
siCCDC106
Ab
MW
CCDC106
180kDa
130kDa
95kDa
70kDa
55kDa
43kDa
33kDa
25kDa

## Slide 2
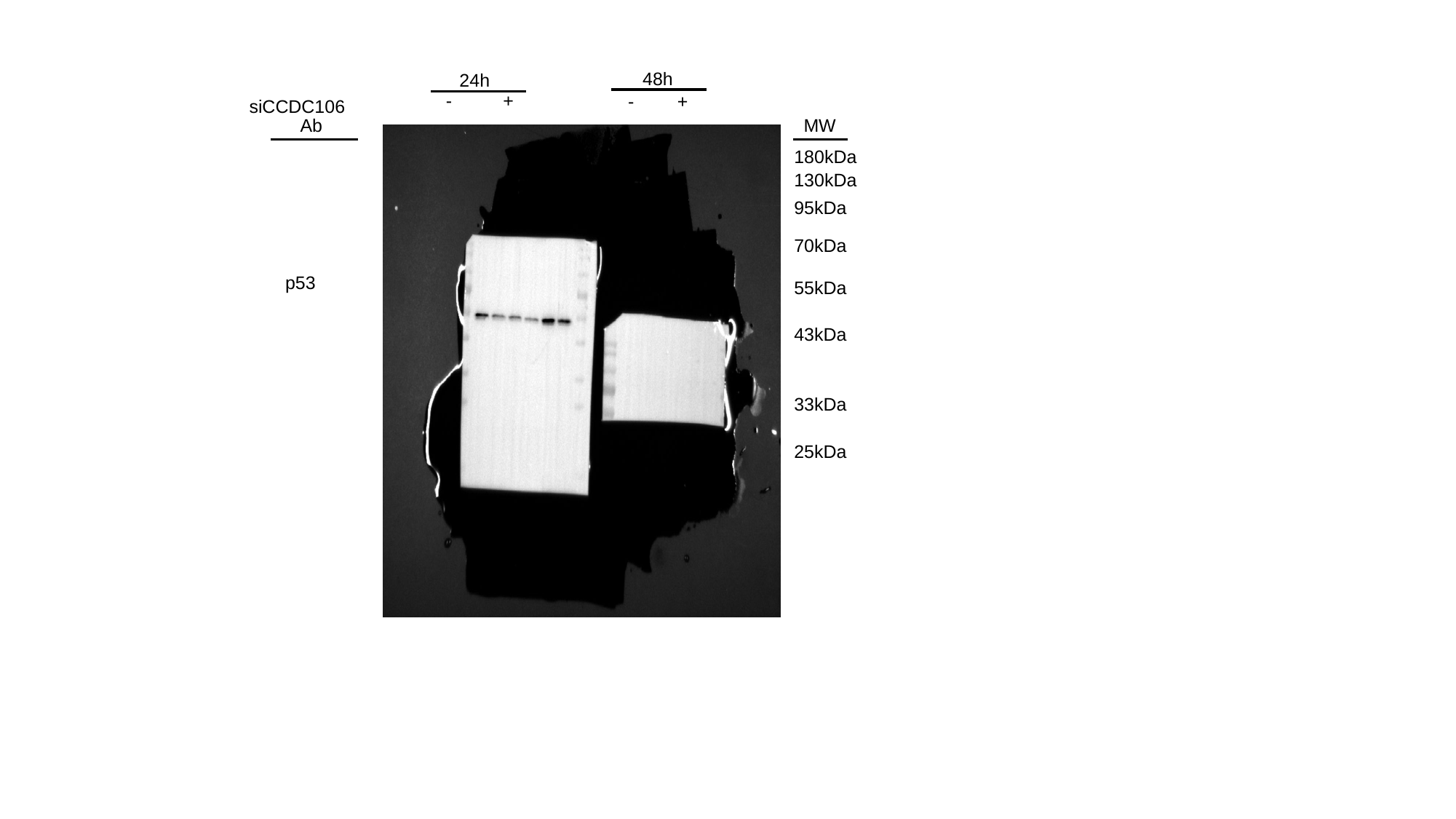

48h
24h
-
+
-
+
siCCDC106
Ab
MW
p53
180kDa
130kDa
95kDa
70kDa
55kDa
43kDa
33kDa
25kDa

## Slide 3
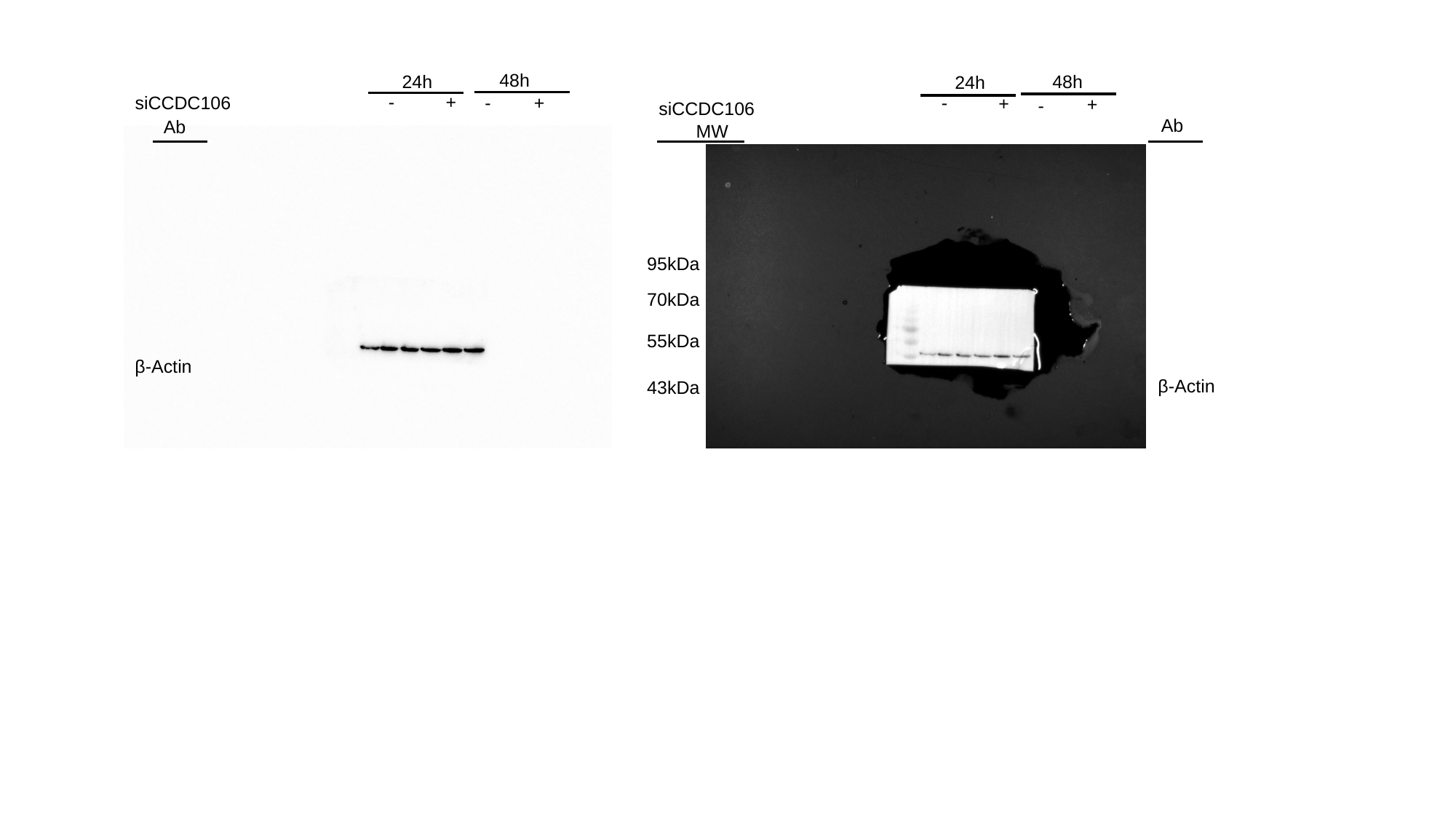

48h
48h
24h
-
+
+
-
siCCDC106
Ab
MW
β-Actin
24h
-
+
-
+
siCCDC106
Ab
95kDa
70kDa
55kDa
β-Actin
43kDa
