# Supplementary material for: Molecular mechanism of CCDC106 regulating the p53-Mdm2/MdmX signaling axis
Source: Sci Rep. 2023 Dec 11;13:21892. doi: 10.1038/s41598-023-47808-z (PMC10713525; doi:10.1038/s41598-023-47808-z)
Supplement: Supplementary file 2 — Supplementary Information 2. [file 41598_2023_47808_MOESM2_ESM.zip › Fig2_3_4/fig2f.pptx]

## Slide 1
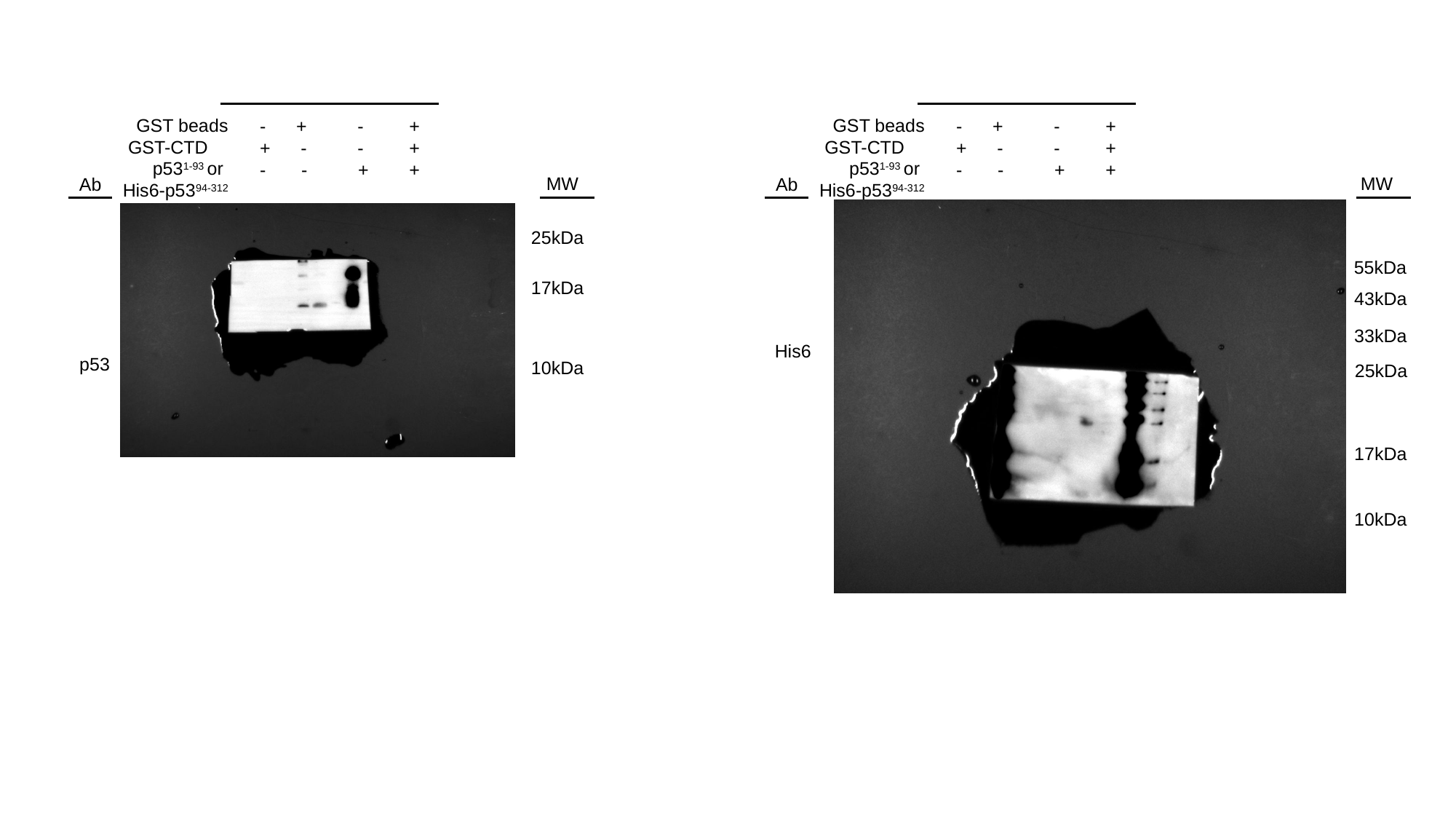

MW
Ab
p53
MW
Ab
His6
GST beads
GST-CTD
p531-93 or
His6-p5394-312
 - + - +
 + - - +
 - - + +
GST beads
GST-CTD
p531-93 or
His6-p5394-312
 - + - +
 + - - +
 - - + +
25kDa
55kDa
17kDa
43kDa
33kDa
10kDa
25kDa
17kDa
10kDa
