# Supplementary material for: Molecular mechanism of CCDC106 regulating the p53-Mdm2/MdmX signaling axis
Source: Sci Rep. 2023 Dec 11;13:21892. doi: 10.1038/s41598-023-47808-z (PMC10713525; doi:10.1038/s41598-023-47808-z)
Supplement: Supplementary file 2 — Supplementary Information 2. [file 41598_2023_47808_MOESM2_ESM.zip › Fig2_3_4/fig2g.pptx]

## Slide 1
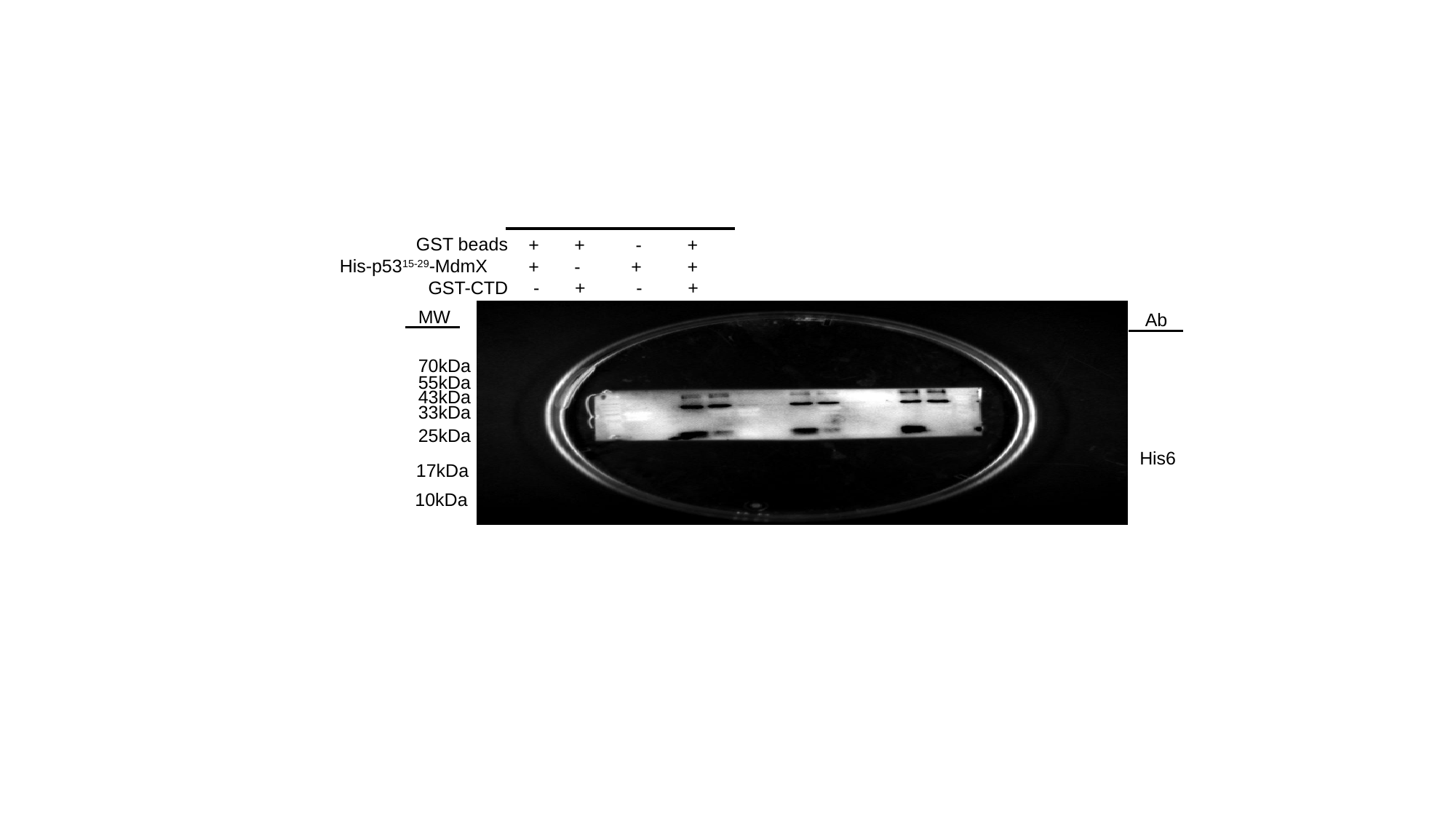

GST beads
His-p5315-29-MdmX
GST-CTD
 + + - +
 + - + +
 - + - +
MW
Ab
His6
70kDa
55kDa
43kDa
33kDa
25kDa
17kDa
10kDa

## Slide 2
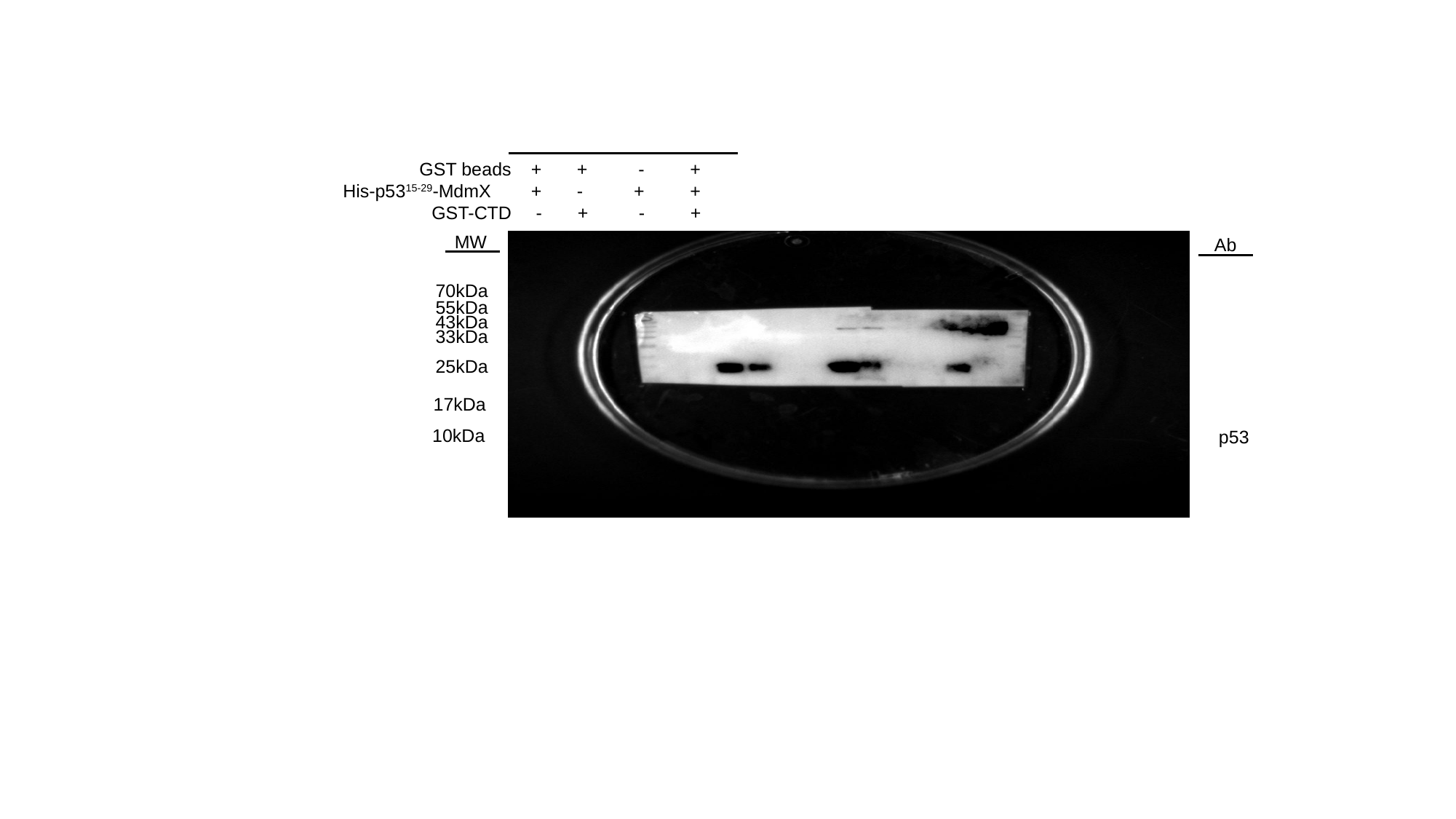

GST beads
His-p5315-29-MdmX
GST-CTD
 + + - +
 + - + +
 - + - +
MW
Ab
p53
70kDa
55kDa
43kDa
33kDa
25kDa
17kDa
10kDa
