# Supplementary material for: Molecular mechanism of CCDC106 regulating the p53-Mdm2/MdmX signaling axis
Source: Sci Rep. 2023 Dec 11;13:21892. doi: 10.1038/s41598-023-47808-z (PMC10713525; doi:10.1038/s41598-023-47808-z)
Supplement: Supplementary file 2 — Supplementary Information 2. [file 41598_2023_47808_MOESM2_ESM.zip › Fig2_3_4/Fig3.pptx]

## Slide 1
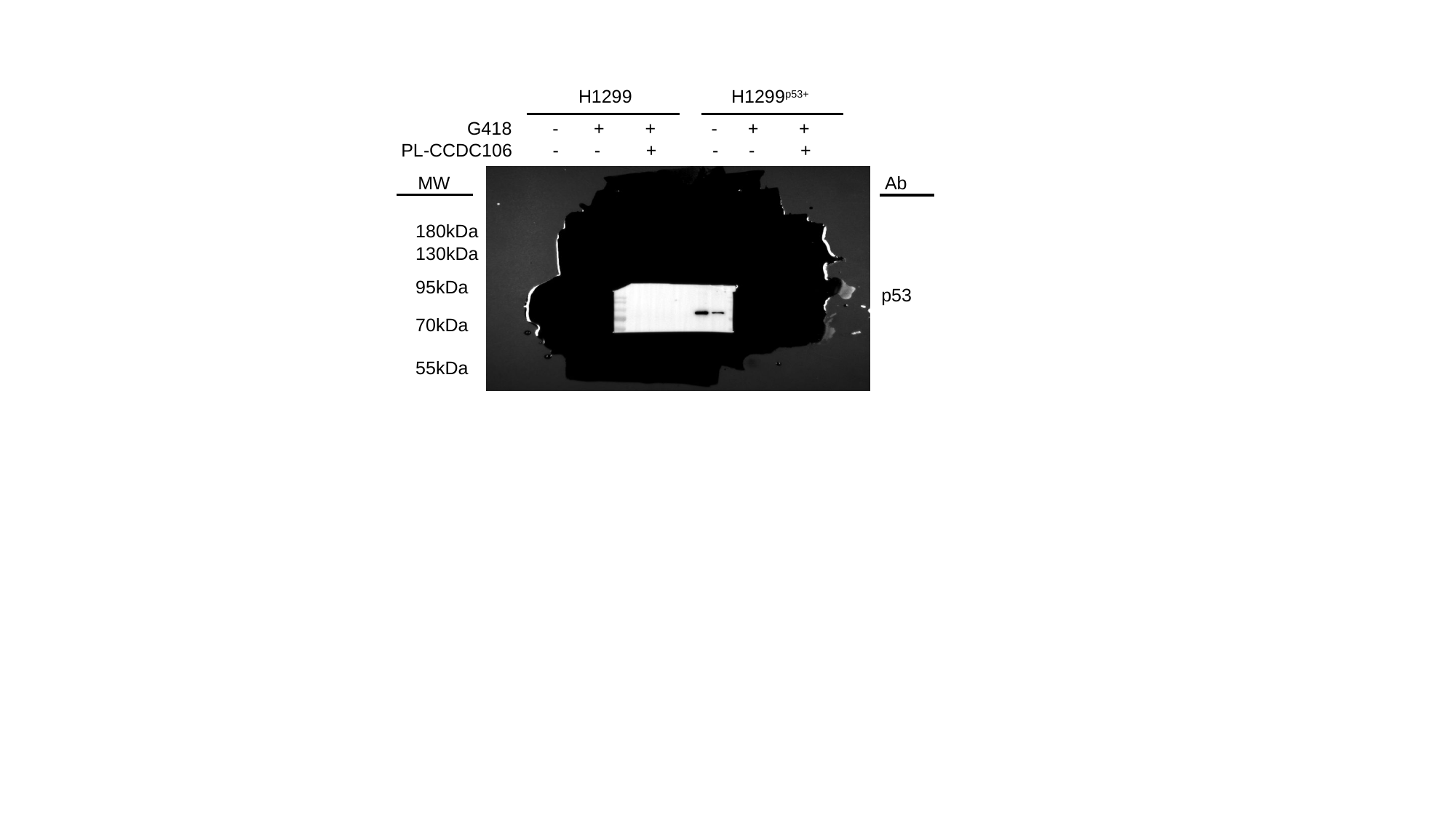

H1299p53+
H1299
 G418 - + + - + +
 PL-CCDC106 - - + - - +
MW
Ab
p53
180kDa
130kDa
95kDa
70kDa
55kDa

## Slide 2
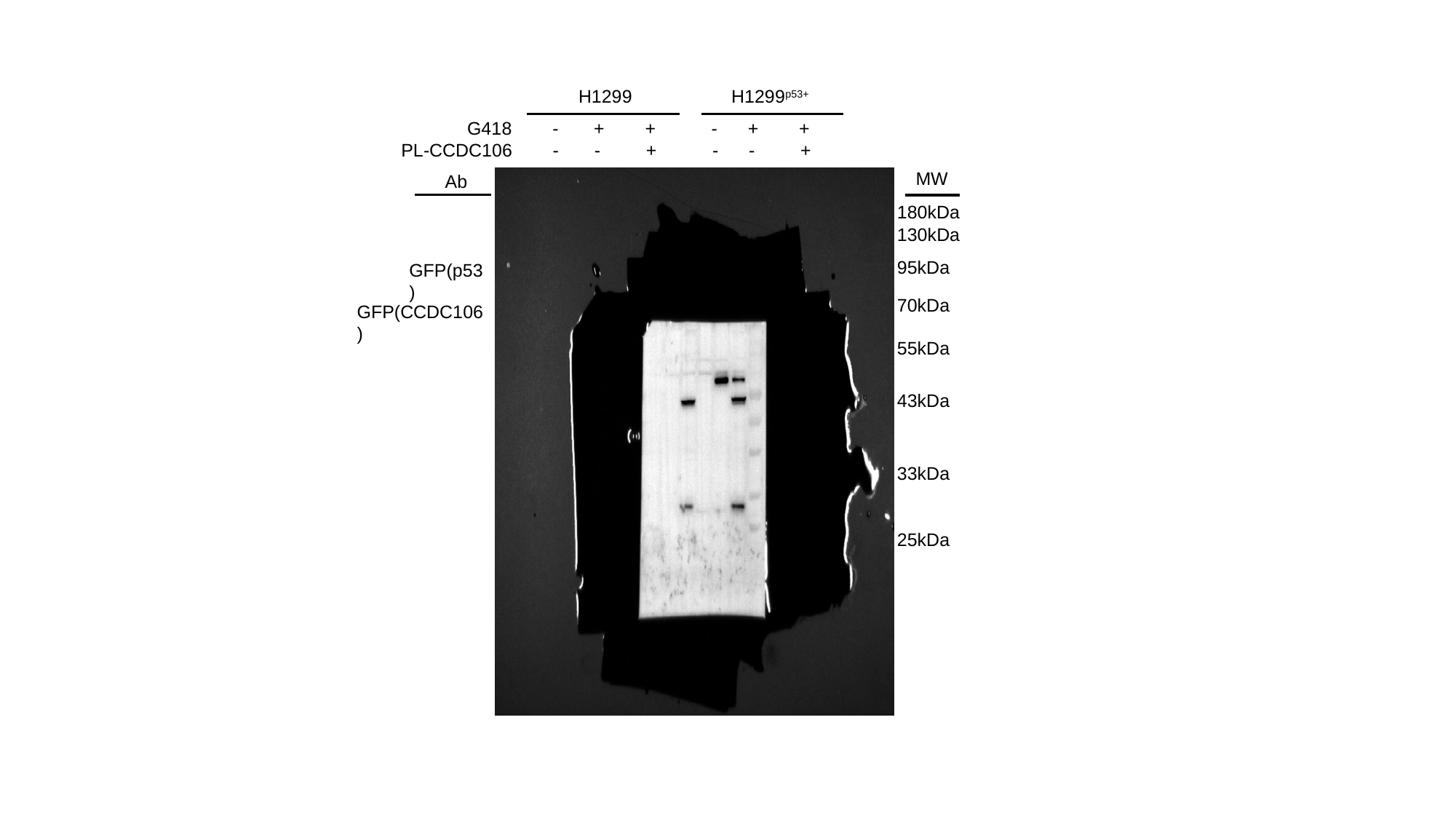

H1299p53+
H1299
 G418 - + + - + +
 PL-CCDC106 - - + - - +
MW
Ab
180kDa
130kDa
95kDa
GFP(p53)
70kDa
GFP(CCDC106)
55kDa
43kDa
33kDa
25kDa

## Slide 3
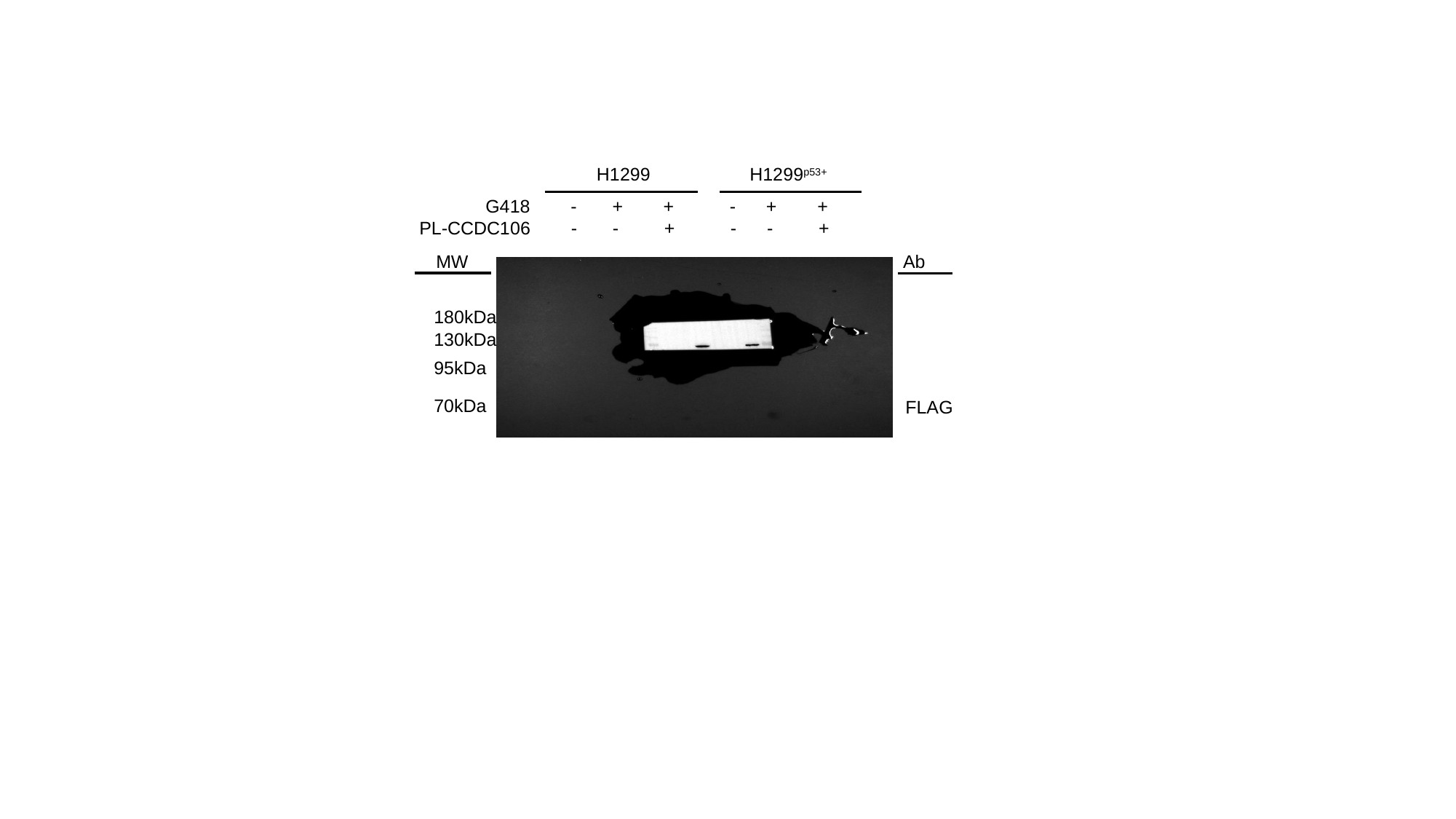

H1299p53+
H1299
 G418 - + + - + +
 PL-CCDC106 - - + - - +
MW
Ab
FLAG
180kDa
130kDa
95kDa
70kDa

## Slide 4
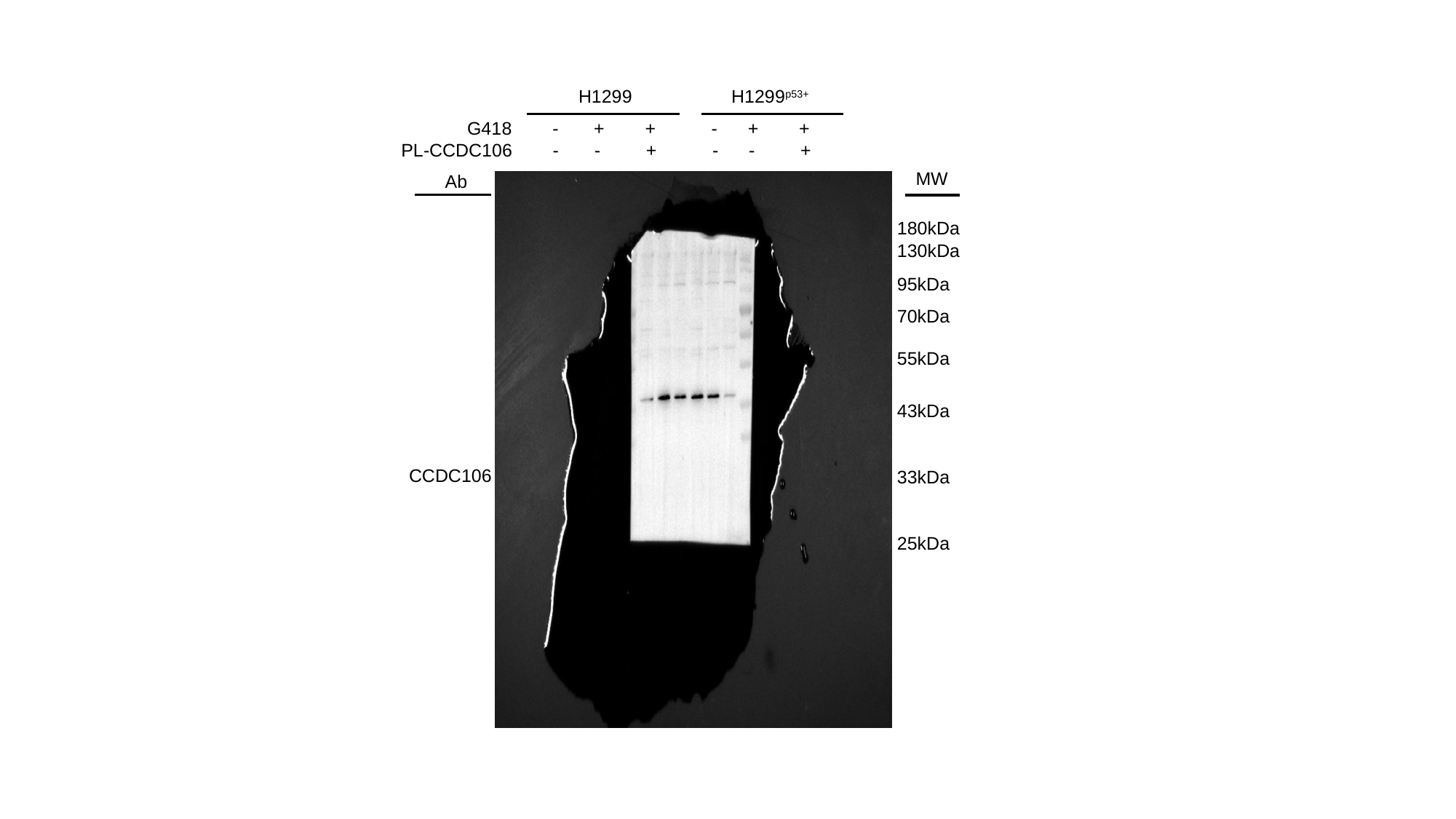

H1299p53+
H1299
 G418 - + + - + +
 PL-CCDC106 - - + - - +
MW
Ab
180kDa
130kDa
95kDa
70kDa
55kDa
43kDa
CCDC106
33kDa
25kDa

## Slide 5
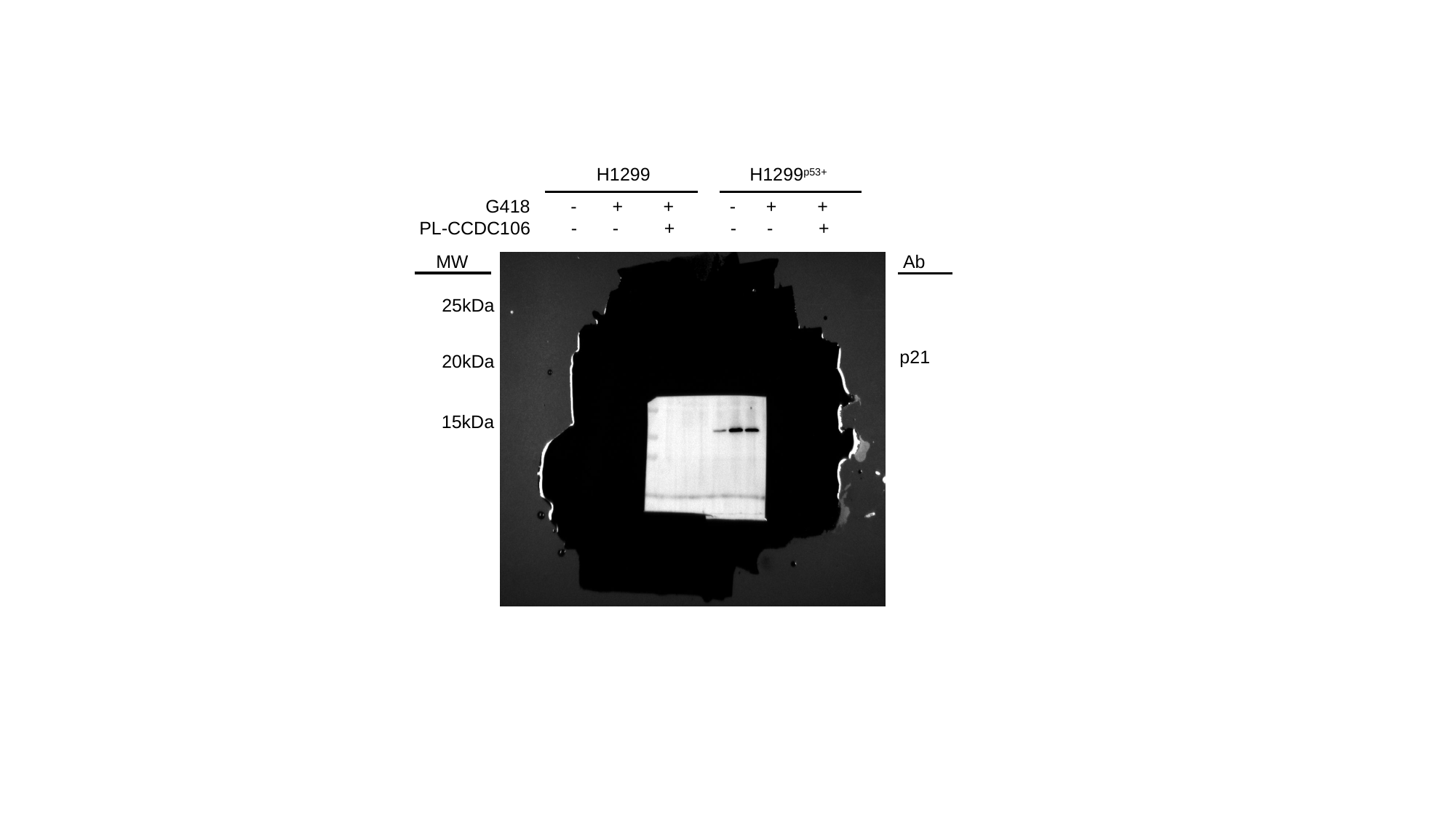

H1299p53+
H1299
 G418 - + + - + +
 PL-CCDC106 - - + - - +
MW
Ab
p21
25kDa
20kDa
15kDa

## Slide 6
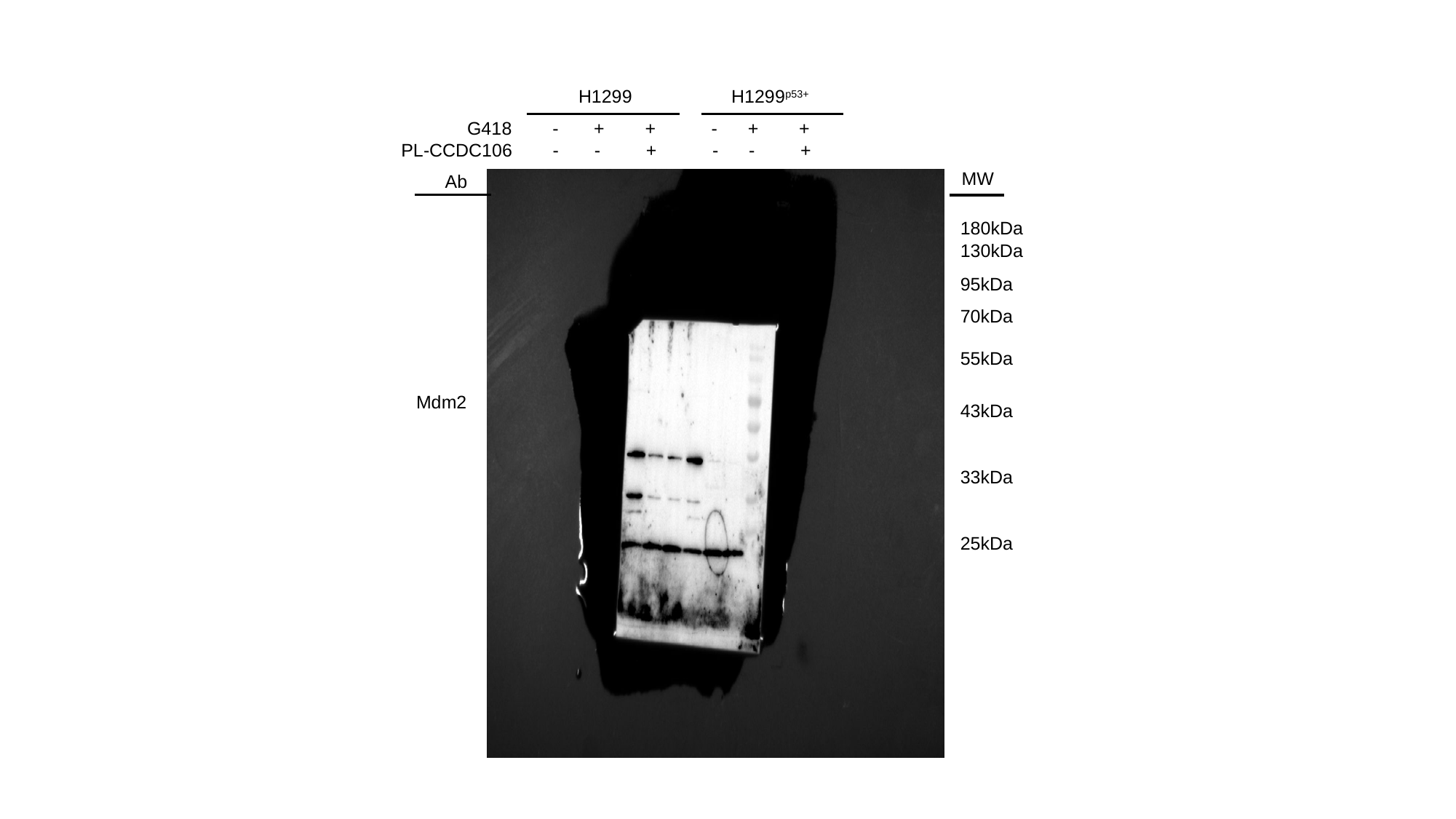

H1299p53+
H1299
 G418 - + + - + +
 PL-CCDC106 - - + - - +
MW
Ab
180kDa
130kDa
95kDa
70kDa
55kDa
Mdm2
43kDa
33kDa
25kDa

## Slide 7
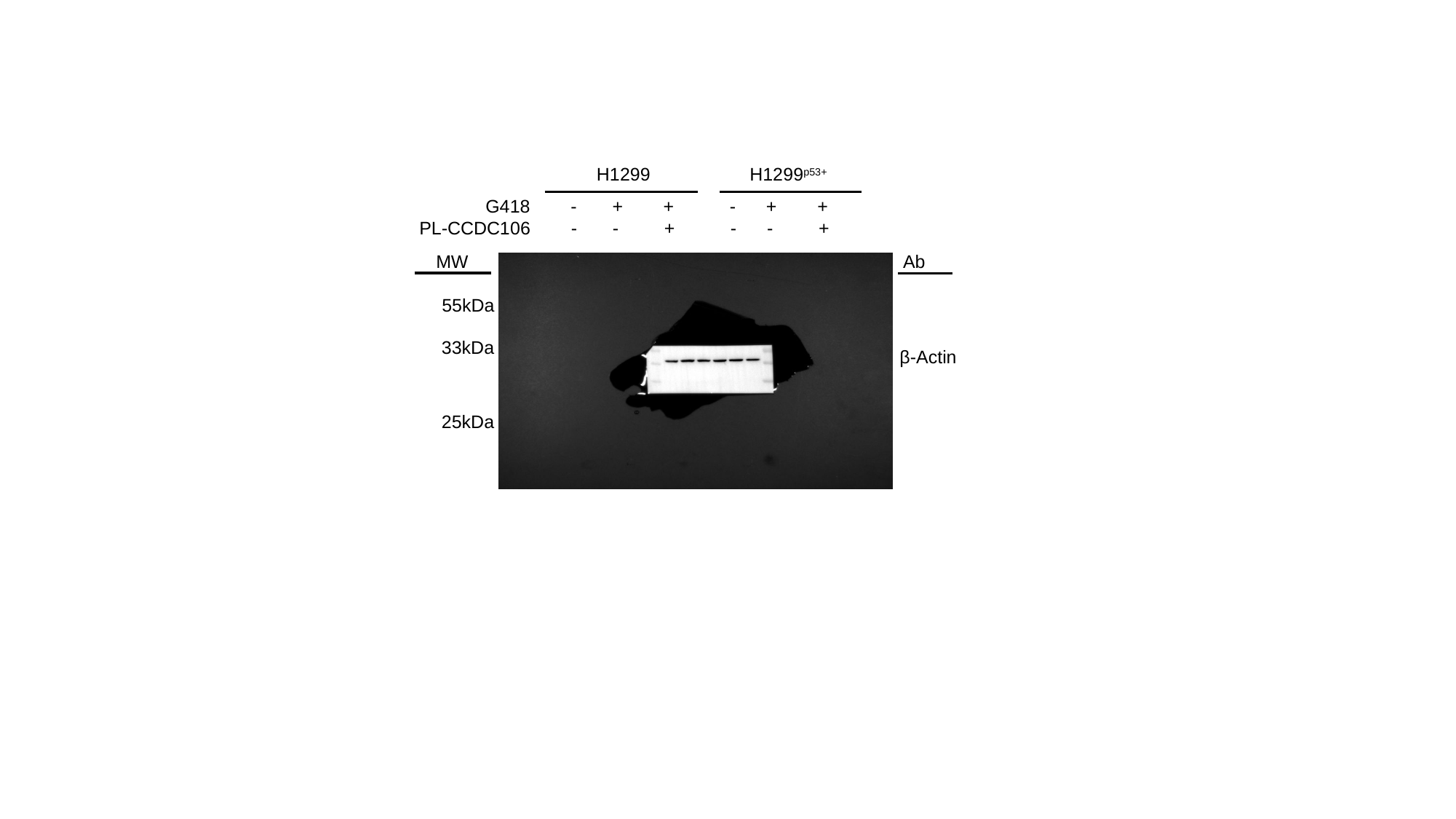

H1299p53+
H1299
 G418 - + + - + +
 PL-CCDC106 - - + - - +
MW
Ab
β-Actin
55kDa
33kDa
25kDa
