# Supplementary material for: Molecular mechanism of CCDC106 regulating the p53-Mdm2/MdmX signaling axis
Source: Sci Rep. 2023 Dec 11;13:21892. doi: 10.1038/s41598-023-47808-z (PMC10713525; doi:10.1038/s41598-023-47808-z)
Supplement: Supplementary file 2 — Supplementary Information 2. [file 41598_2023_47808_MOESM2_ESM.zip › Fig2_3_4/Fig4b.pptx]

## Slide 1
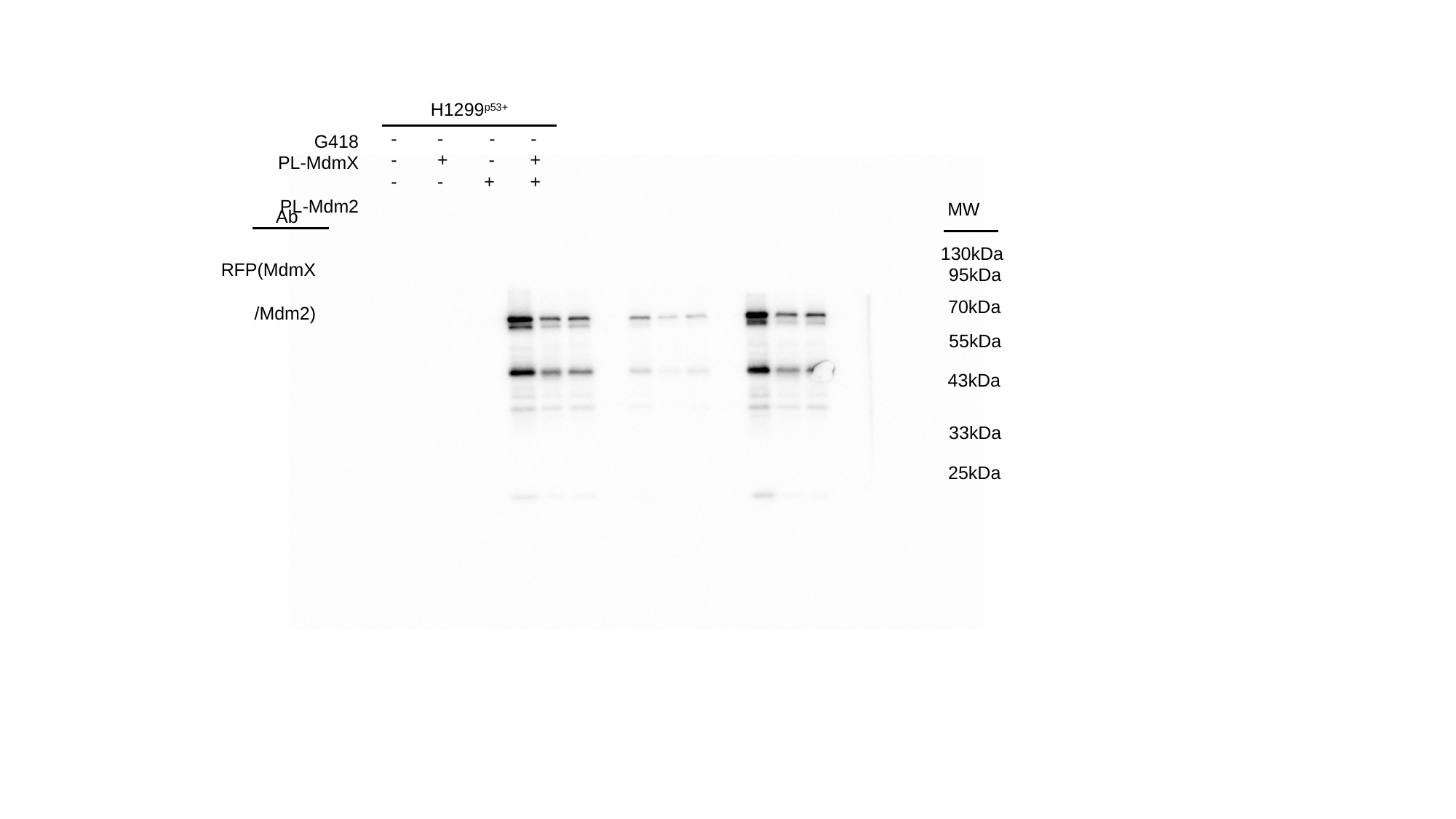

H1299p53+
 - - - -
 - + - +
 - - + +
G418
PL-MdmX
PL-Mdm2
MW
Ab
130kDa
RFP(MdmX
 /Mdm2)
95kDa
70kDa
55kDa
43kDa
33kDa
25kDa

## Slide 2
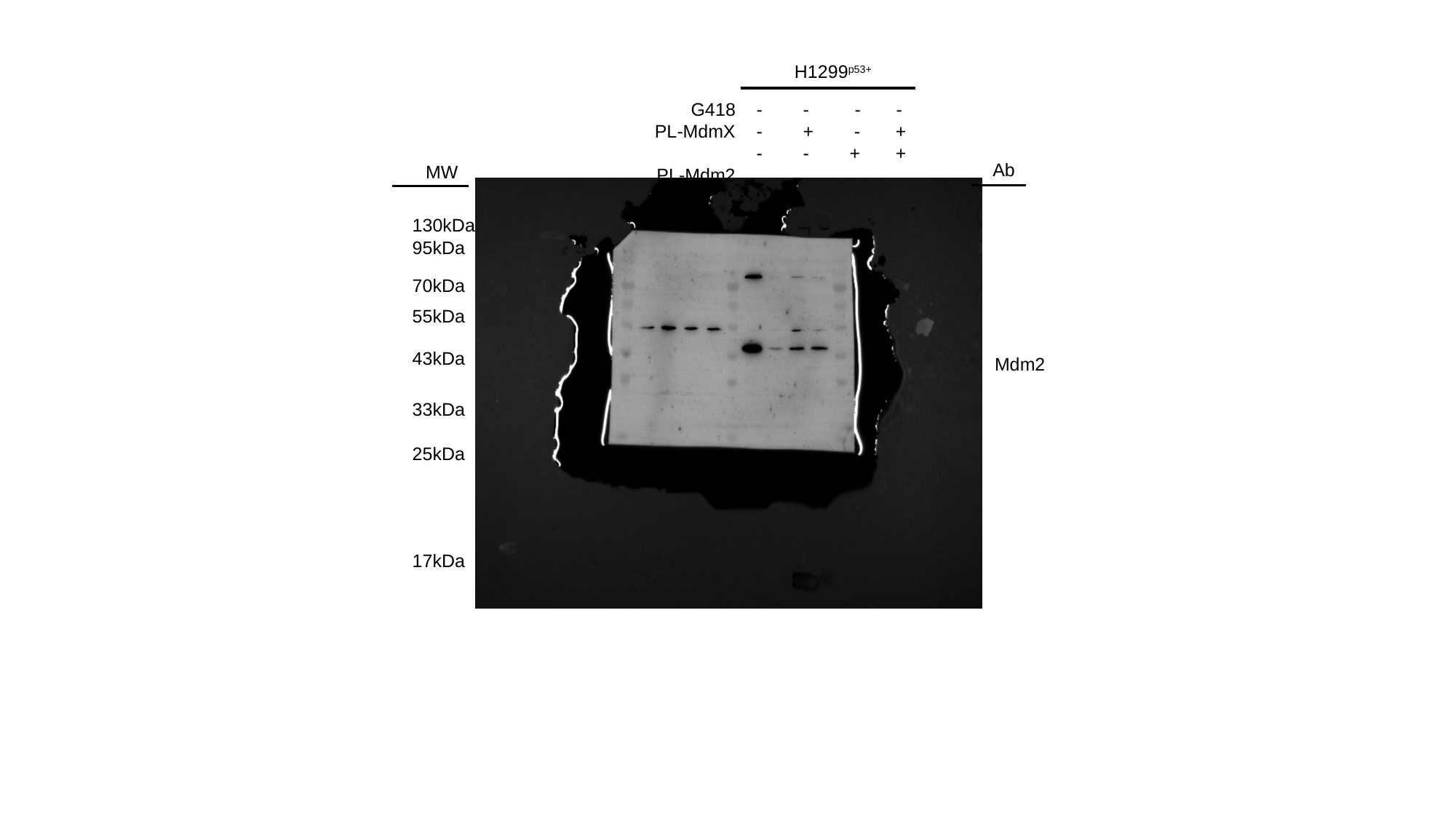

H1299p53+
G418
PL-MdmX
PL-Mdm2
 - - - -
 - + - +
 - - + +
Ab
MW
Mdm2
130kDa
95kDa
70kDa
55kDa
43kDa
33kDa
25kDa
17kDa

## Slide 3
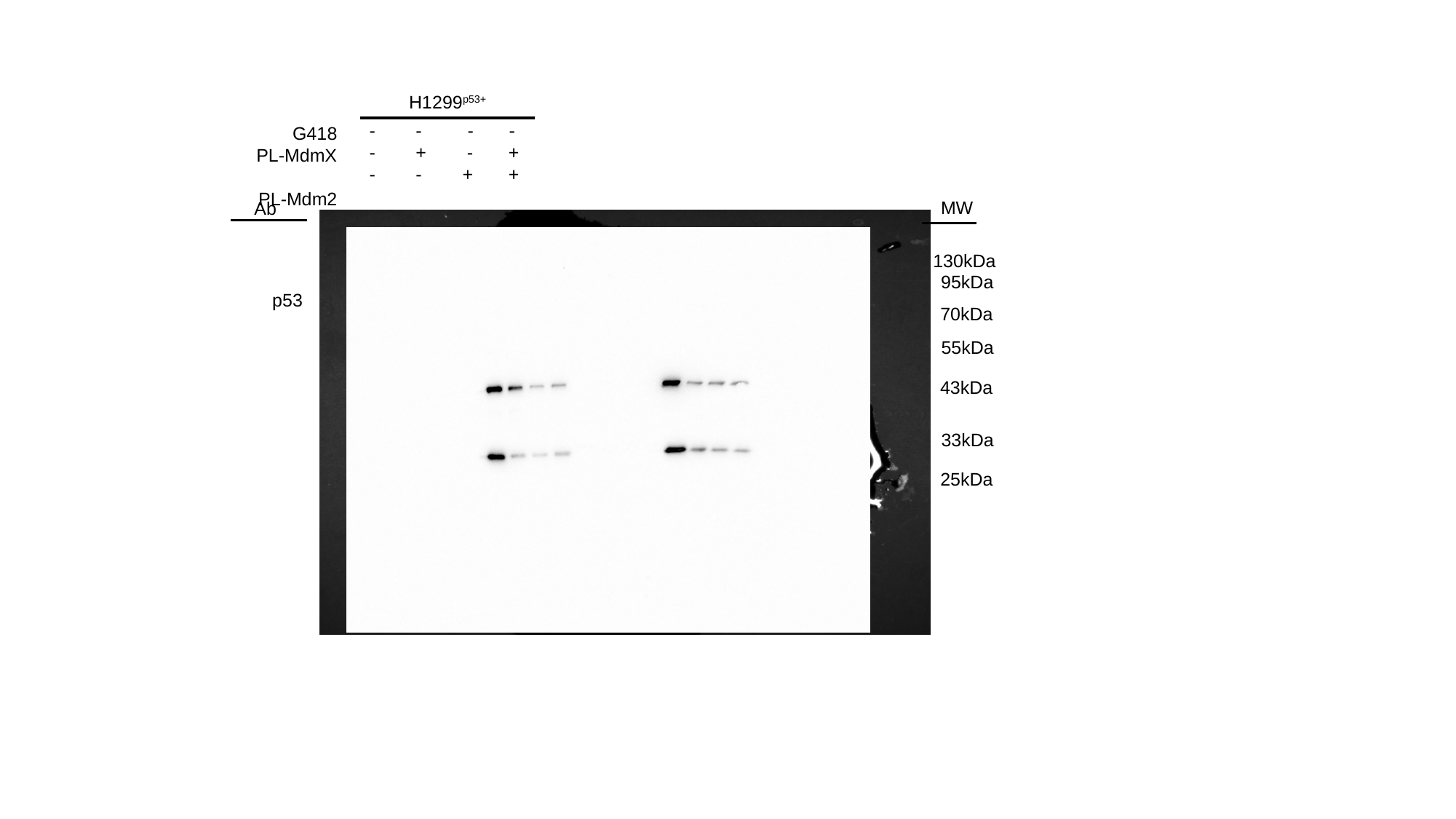

H1299p53+
 - - - -
 - + - +
 - - + +
G418
PL-MdmX
PL-Mdm2
MW
Ab
130kDa
95kDa
p53
70kDa
55kDa
43kDa
33kDa
25kDa

## Slide 4
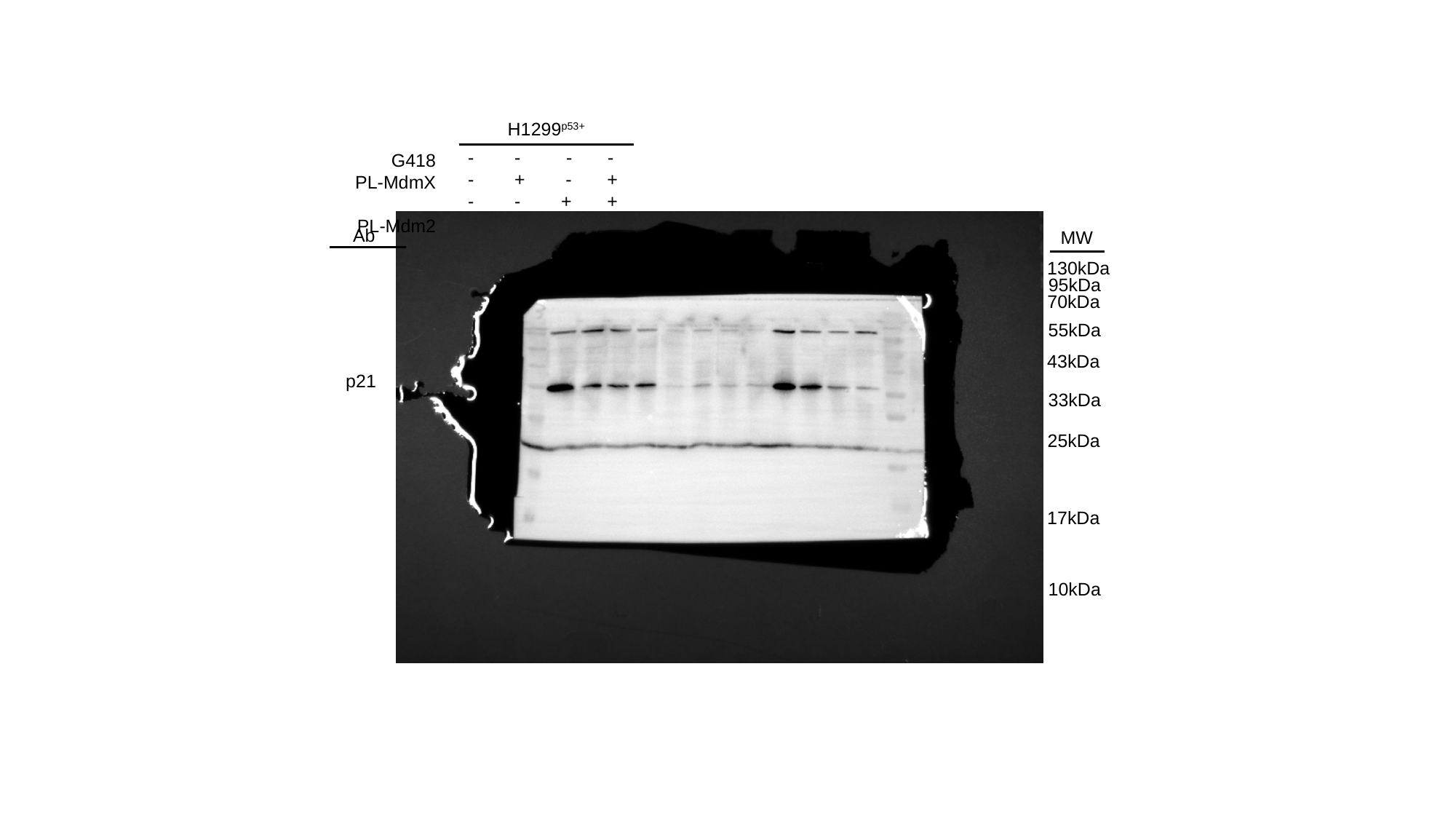

H1299p53+
 - - - -
 - + - +
 - - + +
G418
PL-MdmX
PL-Mdm2
Ab
MW
130kDa
95kDa
70kDa
55kDa
43kDa
p21
33kDa
25kDa
17kDa
10kDa

## Slide 5
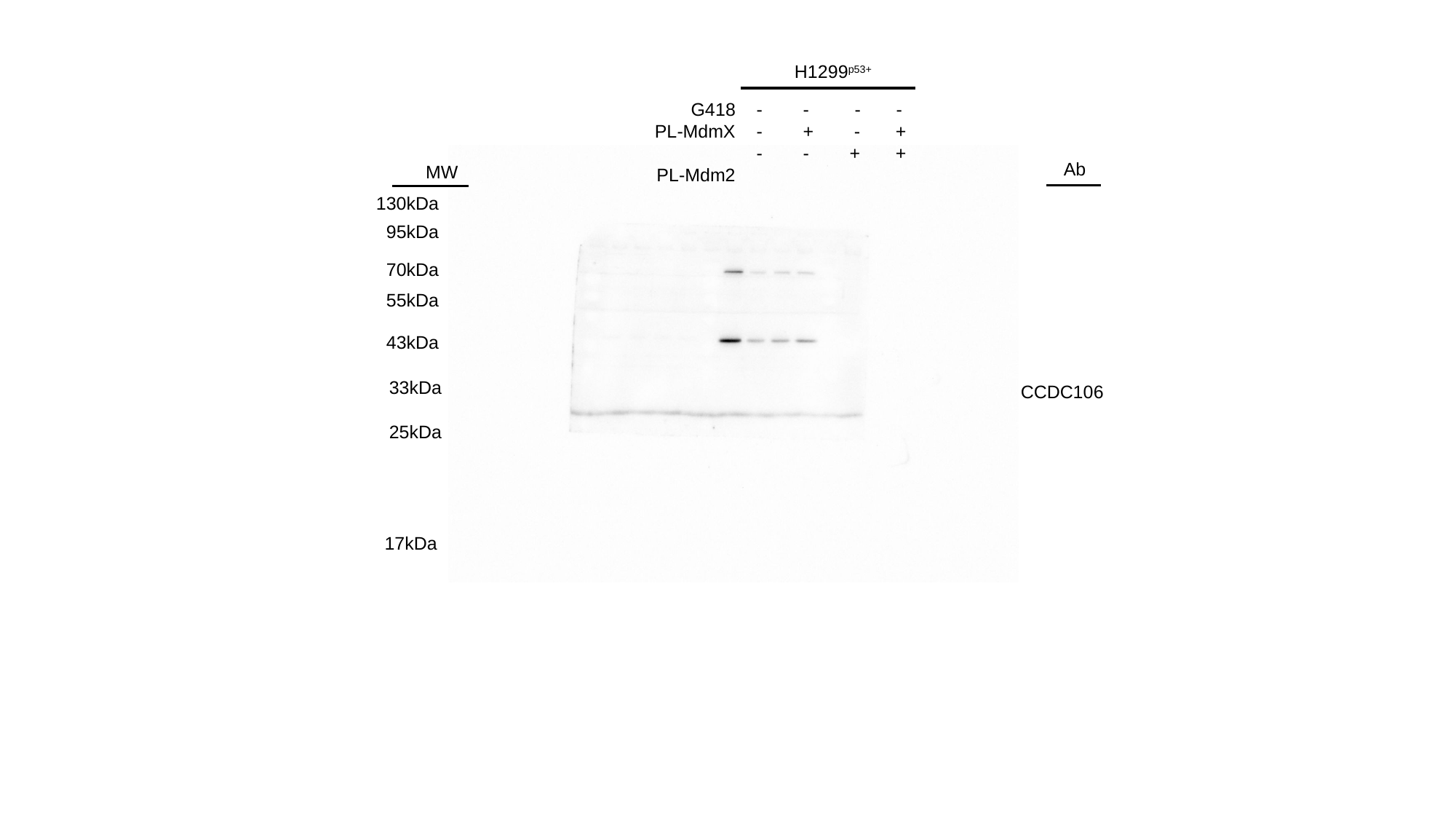

H1299p53+
G418
PL-MdmX
PL-Mdm2
 - - - -
 - + - +
 - - + +
Ab
MW
CCDC106
130kDa
95kDa
70kDa
55kDa
43kDa
33kDa
25kDa
17kDa

## Slide 6
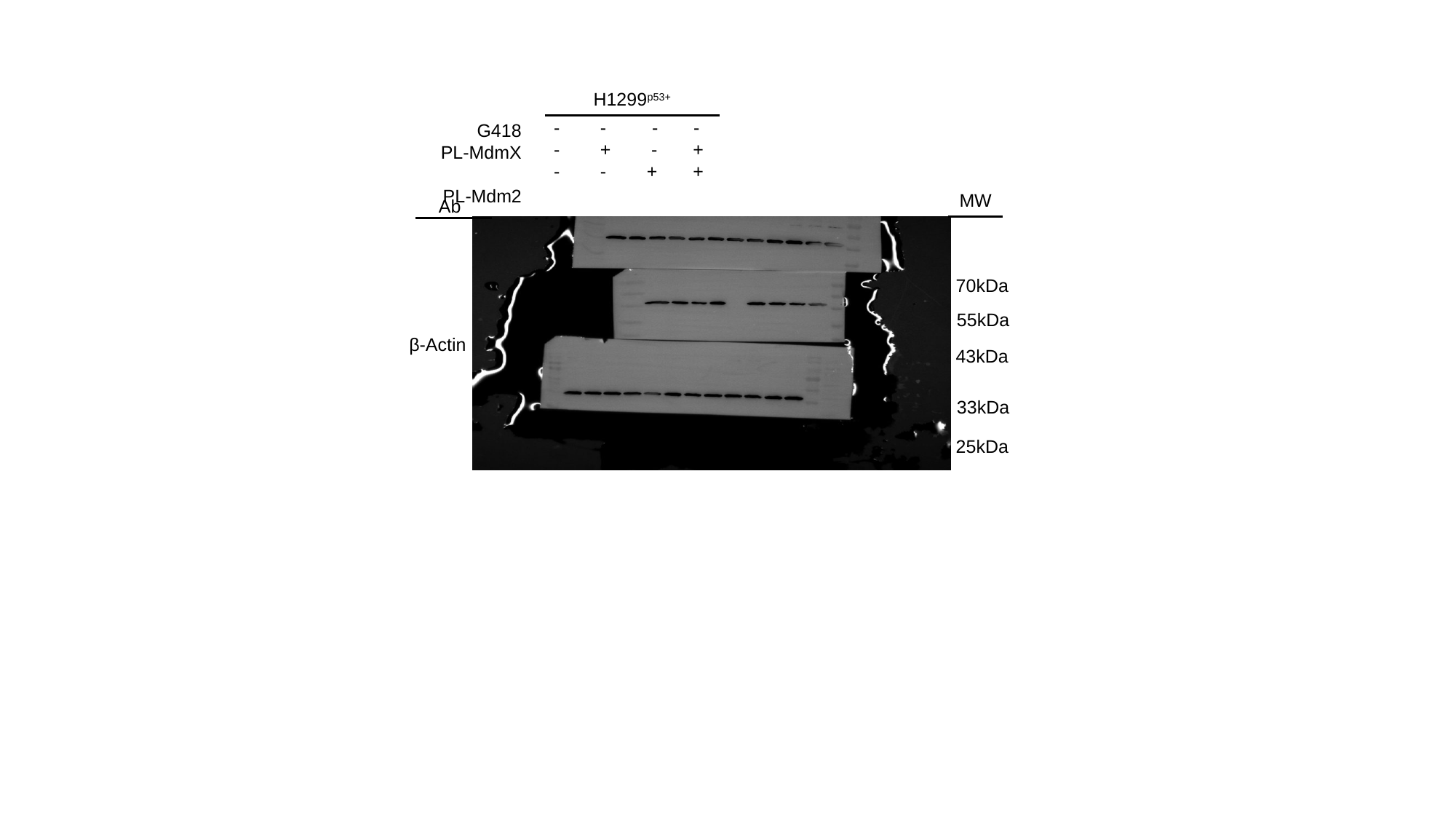

H1299p53+
 - - - -
 - + - +
 - - + +
G418
PL-MdmX
PL-Mdm2
MW
Ab
β-Actin
70kDa
55kDa
43kDa
33kDa
25kDa
