# Supplementary figures and images for: Molecular mechanism of CCDC106 regulating the p53-Mdm2/MdmX signaling axis
Source: Sci Rep. 2023 Dec 11;13:21892. doi: 10.1038/s41598-023-47808-z (PMC10713525; doi:10.1038/s41598-023-47808-z)

## Slide 1
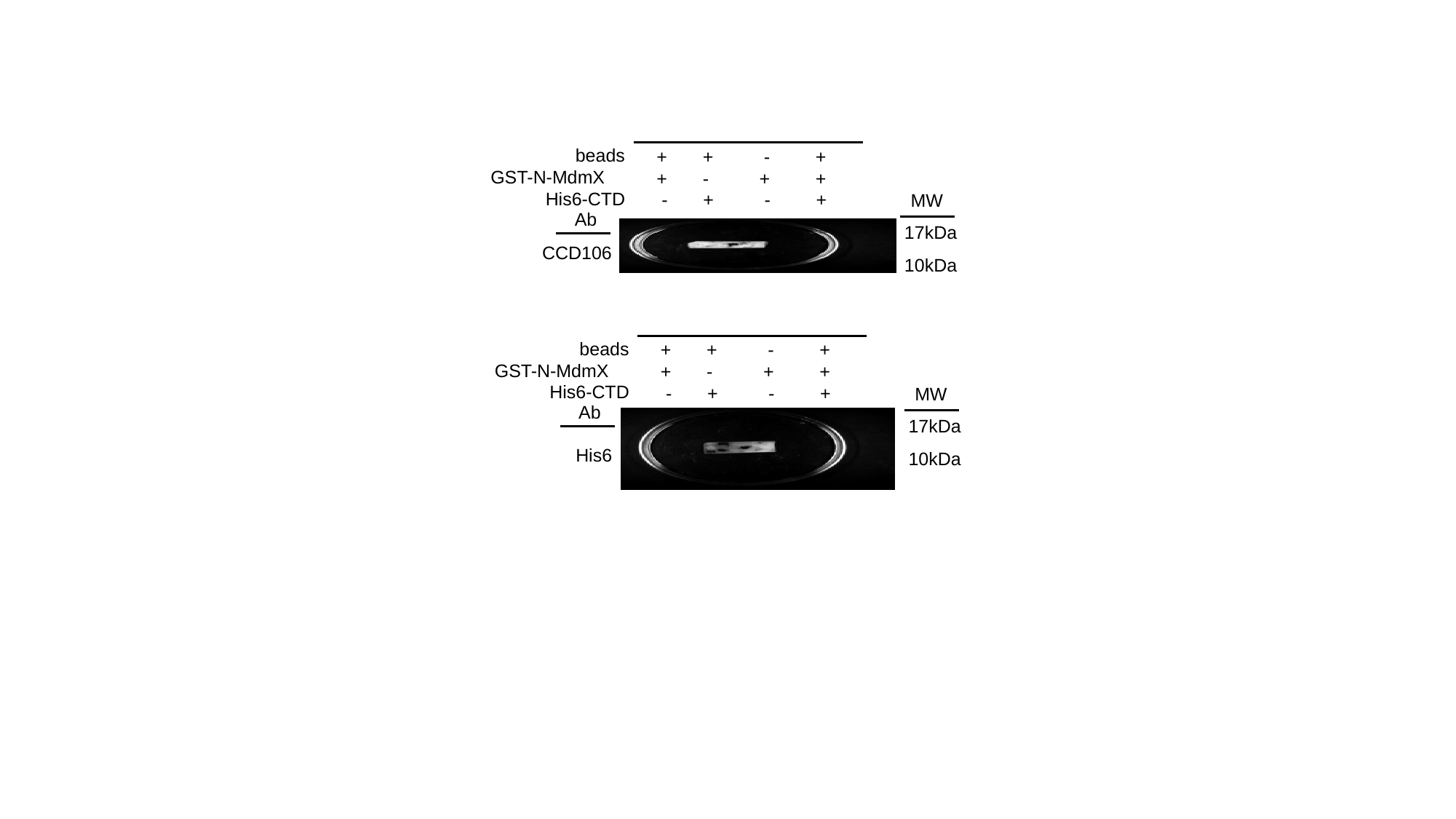

beads
GST-N-MdmX
His6-CTD
 + + - +
 + - + +
 - + - +
MW
Ab
CCD106
17kDa
10kDa
beads
GST-N-MdmX
His6-CTD
 + + - +
 + - + +
 - + - +
MW
Ab
His6
17kDa
10kDa

Supplement: Supplementary file 2 — Supplementary Information 2. [file 41598_2023_47808_MOESM2_ESM.zip › Fig2_3_4/Extended Data Fig. 4.pptx]

## Slide 1
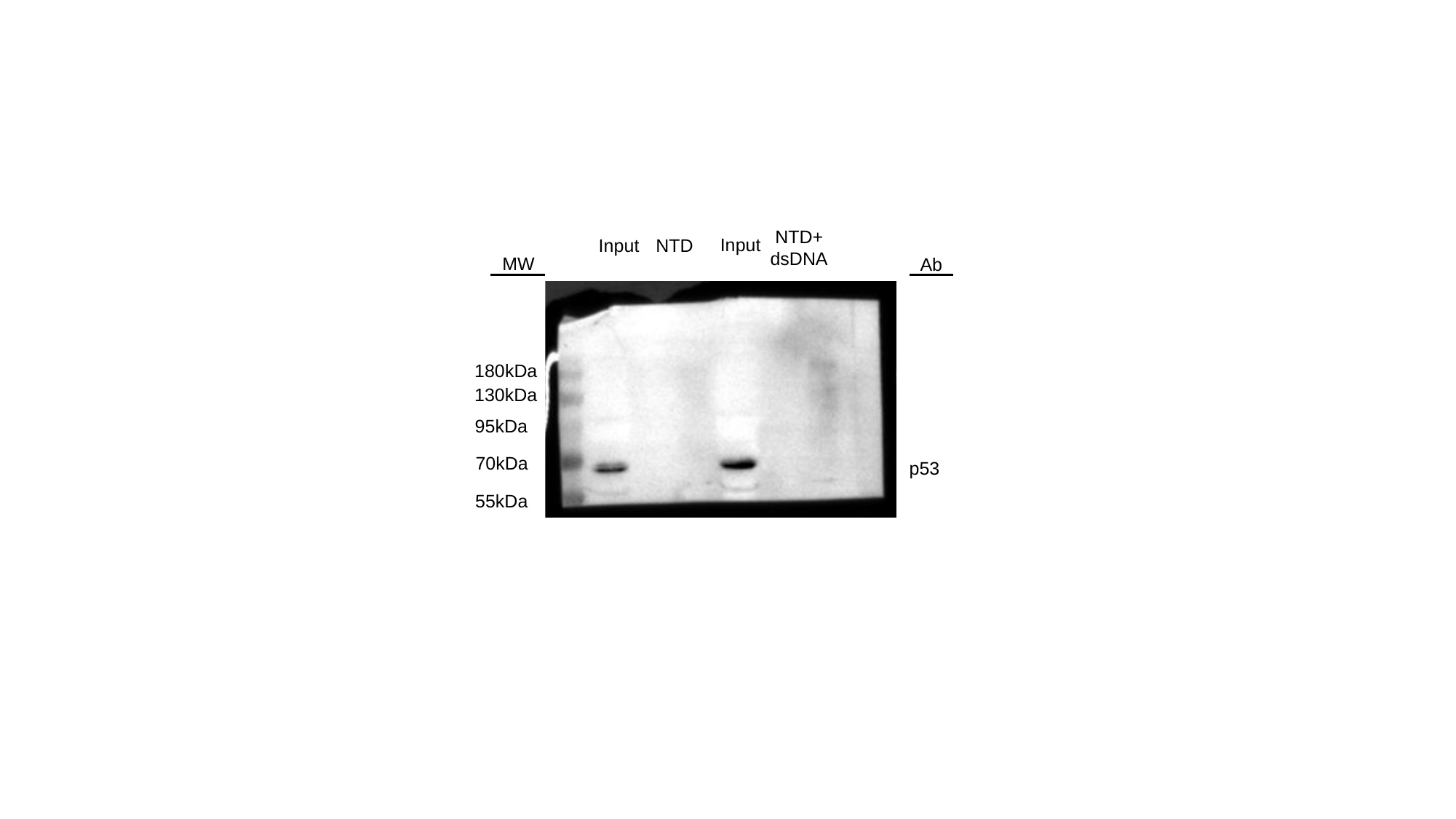

NTD+
dsDNA
Input
Input
NTD
p53
MW
Ab
180kDa
130kDa
95kDa
70kDa
55kDa

Supplement: Supplementary file 2 — Supplementary Information 2. [file 41598_2023_47808_MOESM2_ESM.zip › Fig2_3_4/fig2d.pptx]

## Slide 1
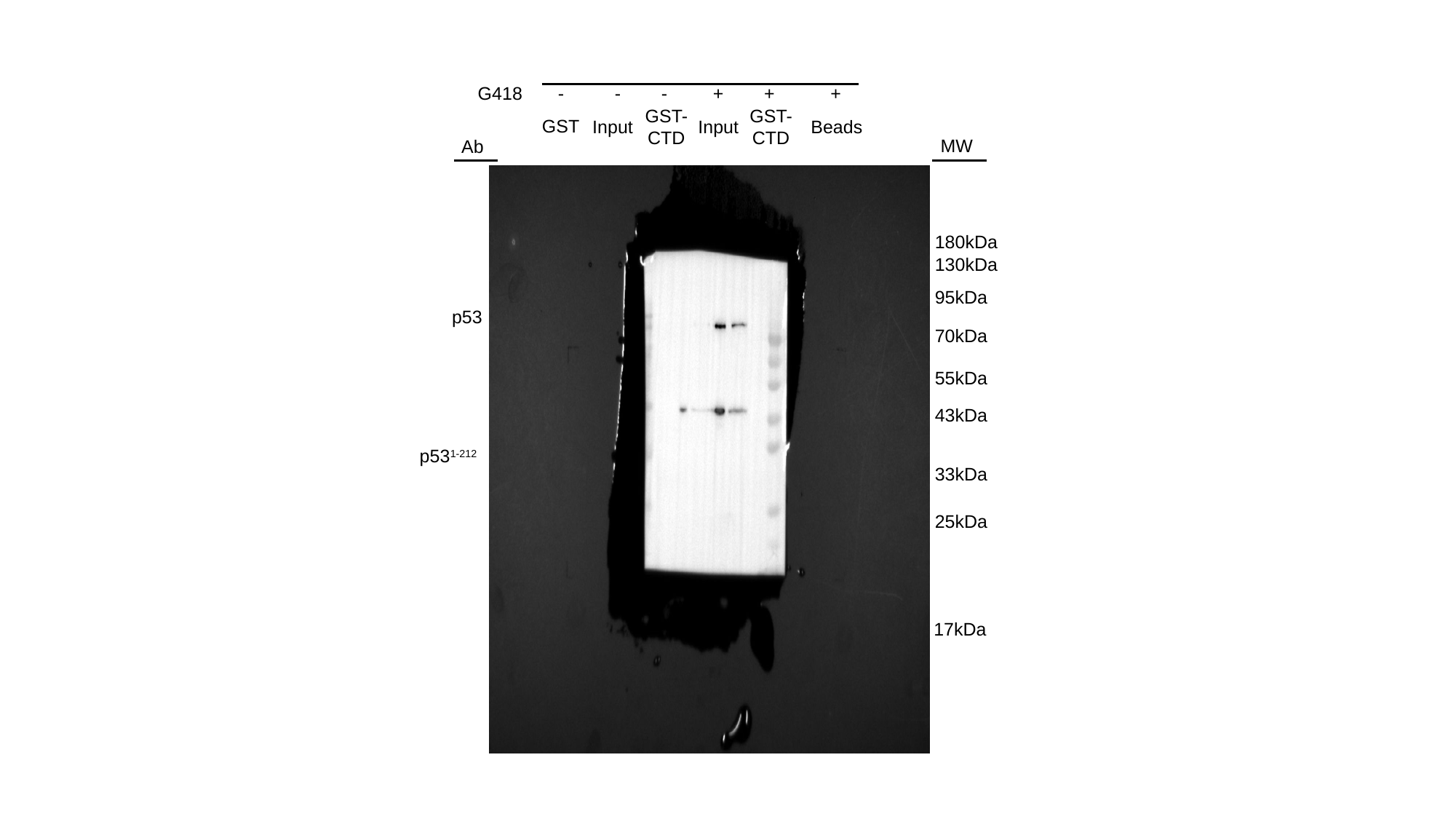

G418 - - - + + +
GST-CTD
GST-CTD
GST
Input
Input
Beads
Ab
p53
p531-212
MW
180kDa
130kDa
95kDa
70kDa
55kDa
43kDa
33kDa
25kDa
17kDa

Supplement: Supplementary file 2 — Supplementary Information 2. [file 41598_2023_47808_MOESM2_ESM.zip › Fig2_3_4/fig2e.pptx]
